# Alternative start codon selection shapes mitochondrial function and rare human diseases

**DOI:** 10.1016/j.molcel.2025.10.013

**Published:** 2025-11-07

**Authors:** Jimmy Ly, Matteo Di Bernardo, Yi Fei Tao, Ekaterina Khalizeva, Christopher J. Giuliano, Sebastian Lourido, Mark D. Fleming, Iain M. Cheeseman

**Affiliations:** 1 Whitehead Institute for Biomedical Research, Cambridge, MA, USA; 2 Department of Biology, Massachusetts Institute of Technology, Cambridge, MA, USA; 3 Department of Pathology, Boston Children’s Hospital, Boston, MA, USA; 4 Lead contact

## Abstract

Rare genetic diseases collectively affect millions of individuals. A common target of many rare diseases is the mitochondria, intracellular organelles that originated through endosymbiosis. Eukaryotic cells require related proteins to function both within the mitochondria and in the host cell. By analyzing N-terminal protein isoforms generated through alternative start codon selection, we identify hundreds of differentially localized isoform pairs, including dual-localized isoforms that are essential for both mitochondrial and host cell function. Subsets of dual mitochondria-localized isoforms emerged during early eukaryotic evolution, coinciding with mitochondrial endosymbiosis. Importantly, we identify dozens of rare disease alleles that affect these alternative protein variants with unique molecular and clinical consequences. Alternative start codon selection can bypass pathogenic nonsense and frameshift mutations, thereby selectively eliminating specific isoforms, which we term isoform-selective alleles (ISAs). Together, our findings illuminate the evolutionary and pathological relevance of alternative translation, offering insights into the molecular basis of rare human diseases.

## In brief

Ly et al. demonstrate that alternative start codon selection generates distinct N-terminal isoforms from a single mRNA, influencing mitochondrial function, evolution, and rare diseases. They uncover mechanisms controlling alternative N-terminal isoform localization and show that disease alleles can selectively disrupt specific isoforms, terming these mutations as “isoform-selective alleles.”

## Graphical Abstract

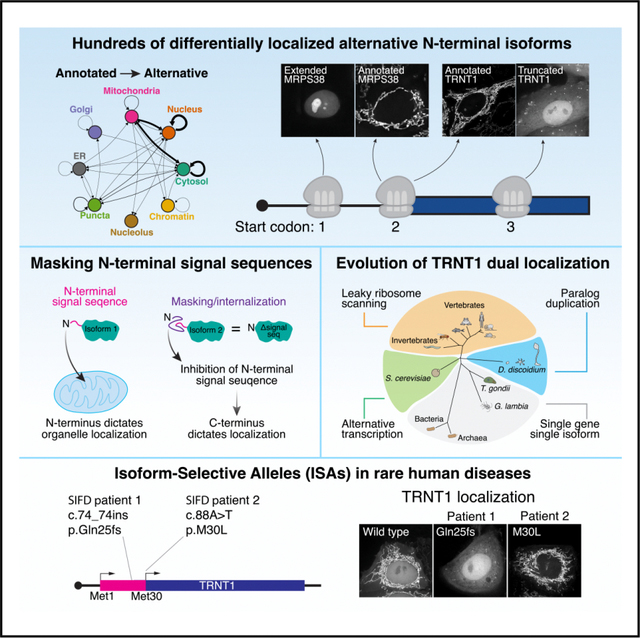

## INTRODUCTION

With over 6,000 rare genetic diseases collectively affecting 3%–5% of the global population, hundreds of millions of individuals are impacted by such diseases worldwide.^1^ To effectively diagnose and treat rare diseases, it is critical to define systematic strategies to evaluate the potential impact of disease mutations on protein and gene function. The analysis of genetic disease alleles is typically based on gene annotations in which the open reading frame (ORF)^2^ is defined as the longest ORF in the gene’s mRNA that begins with an AUG start codon. However, human cells additionally generate diverse protein products through alterations to the mechanisms by which a gene is decoded,^3,4^ including alternative translation. In cases in which a gene is decoded into multiple alternative protein products, it is critical to consider the selective impact of disease alleles on protein variants. Furthermore, variant pathogenicity is typically assessed based on the predicted effects at the protein level, which does not account for the impact of these variants on alternative start codon selection.

Mitochondrial function is critical to eukaryotic cells, and dysregulation of mitochondrial processes has been linked to hundreds of rare human diseases.^5,6^ The evolution of the mitochondria required specialized adaptations in the early eukaryotic ancestor.^7–10^ Core biological processes, including DNA replication, transcription, and translation, must occur within both the mitochondria and in the host cell, requiring the trafficking of the protein machinery involved in these processes to multiple, distinct cellular compartments. To ensure the proper localization of essential activities within mitochondria, most proteins are directed via a primary import pathway that relies on mitochondrial targeting signals present at the N terminus of the protein.^11,12^ These signals facilitate the translocation of unfolded proteins into the mitochondria through the translocase of the outer membrane (TOM) complex.^11^ The import of proteins into the mitochondria is typically irreversible such that protein molecules imported into the mitochondria cannot re-localize to other organelles. This creates a substantial challenge when a protein is also required within the cell cytoplasm or nucleus. One evolutionary strategy to achieve similar protein activities in each compartment is through a gene duplication event, followed by the acquisition of a mitochondrial targeting signal for one paralog.^7,9,13^ For example, topoisomerase activity is crucial for preventing supercoiling during DNA replication for both mitochondrial and nuclear DNA. In humans, this function is carried out by DNA topoisomerase I (*TOP1*) and DNA topoisomerase I mitochondrial (*TOP1MT*), highly related genes that share 85% protein sequence similarity. TOP1 localizes to the nucleus, whereas TOP1MT localizes to the mitochondria through its acquired mitochondrial targeting signal.^14^ Instead of a gene duplication event, the alternative decoding of a single gene by controlling transcription, splicing, or translation has the potential to produce multiple protein isoforms, including those with differing localization.^15–22^ In particular, protein isoforms with distinct N-terminal sequences would be particularly likely to affect mitochondrial targeting, protein secretion, and other subcellular trafficking events that rely on signal sequences.^23^ However, our understanding of the molecular mechanisms that generate proteins capable of functioning across multiple cellular locations remains limited.

Human cells generate significant proteomic diversity through alternative start codon selection, with an estimated 50% of mRNAs producing more than one protein product.^24–27^ Mechanisms such as leaky ribosome scanning,^19,28,29^ internal ribosome entry,^30,31^ initiation at near-cognate start codons,^3,16,32,33^ and upstream open reading frame (uORF) reinitiation,^34,35^ among others,^36–38^ can each drive the use of alternative start codons within a single mRNA. Downstream translation initiation that is in-frame with the annotated start codon will result in N-terminally truncated protein products. Furthermore, although the region upstream of an annotated start codon is typically described as a 5′ untranslated region (5′ UTR), these sequences can also encode functional protein products. The presence of in-frame alternative start codons in the 5′ UTR, including non-canonical start codons such as CUG and GUG, can produce N-terminal extensions to the annotated protein. However, the widespread functional significance and roles of these alternative isoforms, particularly in the context of rare diseases, remain largely unexplored.

This study uncovers alternative start codon selection as a critical mechanism underlying rare human diseases and shaping mitochondrial function and evolution. Through a systematic analysis of alternative N-terminal variants generated in human cells, we show that these isoforms frequently exhibit distinct subcellular localization compared with their annotated counterparts, with a substantial fraction exhibiting altered mitochondrial localization. We trace the evolutionary origins of alternative start codon selection to primitive eukaryotes following mitochondrial endosymbiosis. Finally, we identify multiple rare disease alleles that selectively affect a single isoform of a gene, including for dual-localized protein isoforms. Our findings suggest that these isoform-selective alleles (ISAs) may contribute to distinct clinical symptoms between patients and should be carefully considered when interpreting genetic variants in rare diseases. These findings highlight the essential role of alternative translation initiation in understanding mutations linked to rare human diseases and offer insights into the evolution and molecular underpinnings of mitochondrial biology.

## RESULTS

### Pervasive changes in the localization of alternative N-terminal isoforms

**Figure 1.**
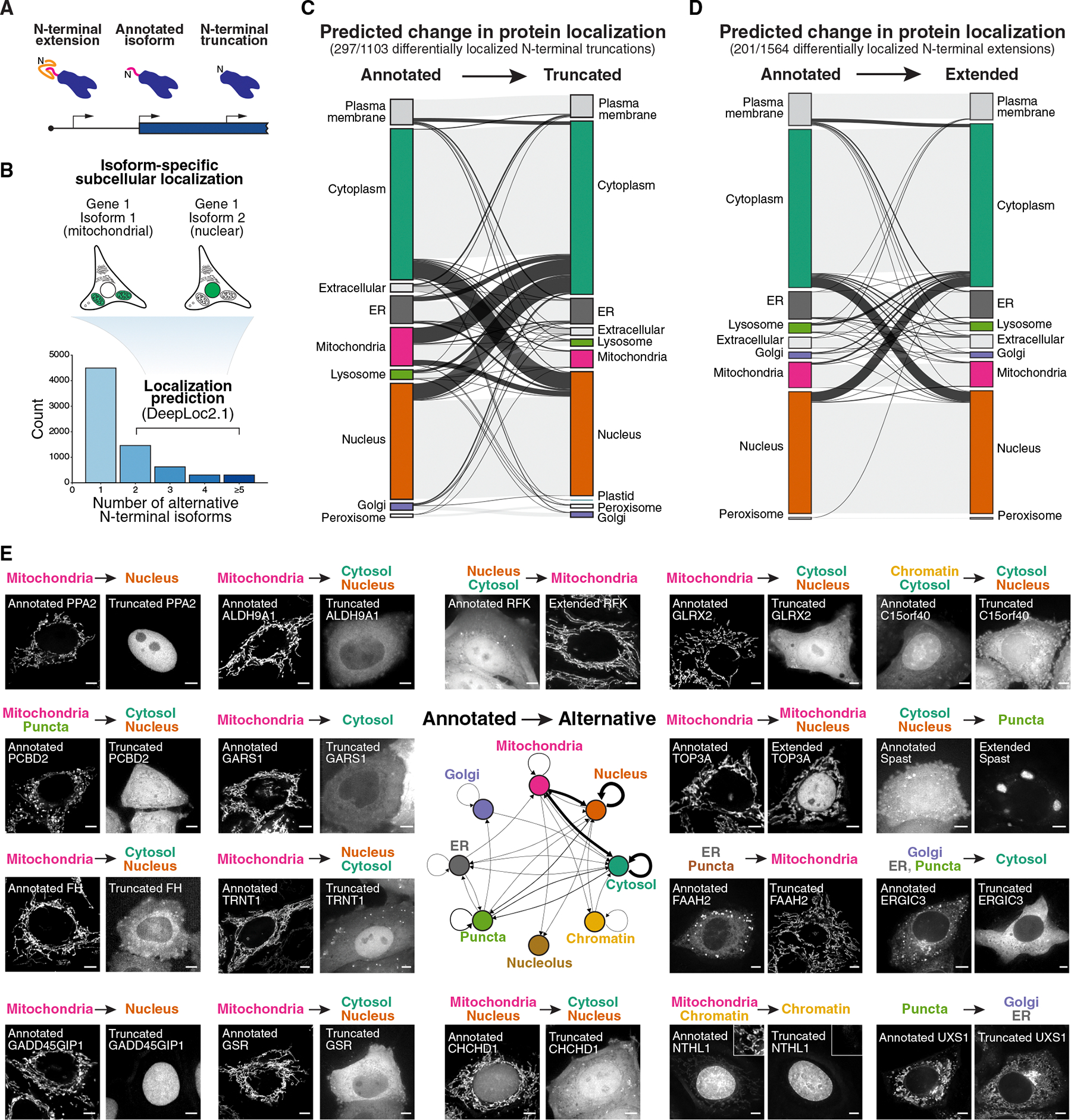
Pervasive differential localization of alternative N-terminal isoforms (A) Schematic of alternative start codon selection as a mechanism to generate proteins with distinct N termini. (B) Histogram showing the number of N-terminal isoforms per gene. For genes with more than one N-terminal isoform, we used DeepLoc2.1 to compare the localization of the annotated, truncated, and extended isoforms. (C) Sankey plot showing the change in predicted subcellular localizations between the annotated isoform and N-terminal truncation based on DeepLoc2.1. The connecting black lines represent predicted changes in localization between annotated and truncated protein isoforms. Faint colored lines indicate no pairs of isoforms that have the same predicted localization. The thickness of the lines represents relative frequency. (D) As described in (C), except depicting the localization changes between the annotated and N-terminal extension. (E) Live-cell images of indicated genes from the alternative N-terminal isoform localization screen. A summary of the differential localization of alternative N-terminal isoforms is shown in the localization network graph in the middle. The line thickness represents increased frequency of this change in localization between isoforms of a gene. Inset images for NTHL1 are scaled to be brighter than the full-size image. Scale bar, 5 μm.

To define the contributions of alternative translational isoforms that differ from the annotated isoform by their N terminus (Figure 1A), we sought to systematically explore the functional differences between annotated proteins and these alternative N-terminal isoforms. Using ribosome profiling, our recent work empirically defined translation initiation sites in human HeLa cells, identifying 15,903 initiation sites in 7,736 genes^39^ (Figures S1A–S1C). Of the identified genes, 2,697 genes produce two or more alternative N-terminal isoforms, including inframe N-terminal extensions or truncations (Figure 1B). As a protein’s N terminus is often critical to direct its subcellular localization, we evaluated genes with two or more protein isoforms for differences in localization signal sequences, including mitochondrial targeting sequences and signal peptides for protein secretion (Figure 1B).

We used DeepLoc2.1, a subcellular localization prediction algorithm based on protein language models,^40^ to predict changes in localization between alternative N-terminal isoforms of a gene (Figures 1B, S1D, and S1E; Table S1; STAR Methods). Among 1,103 pairs of annotated proteins and N-terminally truncated isoforms, 297 (26.9%) were predicted to display differential localization (Figure 1C). Similarly, of 1,564 pairs of annotated proteins and N-terminal extensions, 201 (12.9%) were predicted to show distinct localization patterns (Figure 1D). To validate the differential localization of alternative translational isoforms experimentally, we generated pairs of tagged constructs for 40 genes, tagging either the annotated coding sequence or the alternative isoform with a C-terminal GFP (Figures 1E, S1F, S1G, and S2A). Of the 40 pairs of translational isoforms tested, 27 pairs showed differential subcellular localization (Figures 1E, S1G, and S2A; Table S2). For several selected genes, we also tested whether production of dual-localized proteins is observed when expressing the endogenous mRNA with a C-terminal GFP. Ectopic expression of constructs containing the 5′ UTR-annotated ORF-GFP for tRNA nucleotidyl transferase 1 (TRNT1), aurora kinase A interacting protein 1 (AURKAIP1/MRPS38), glycyl-tRNA synthetase 1 (GARS1), and pyridoxamine 5’-phosphate oxidase (PNPO) each resulted in dual mitochondrial and nuclear/nucleolar localization (Figures S2B–S2G). Thus, alternative translation initiation is a pervasive mechanism for generating dual-localized protein products.

### N-terminal masking of signal sequences acts to regulate protein localization

**Figure 2.**
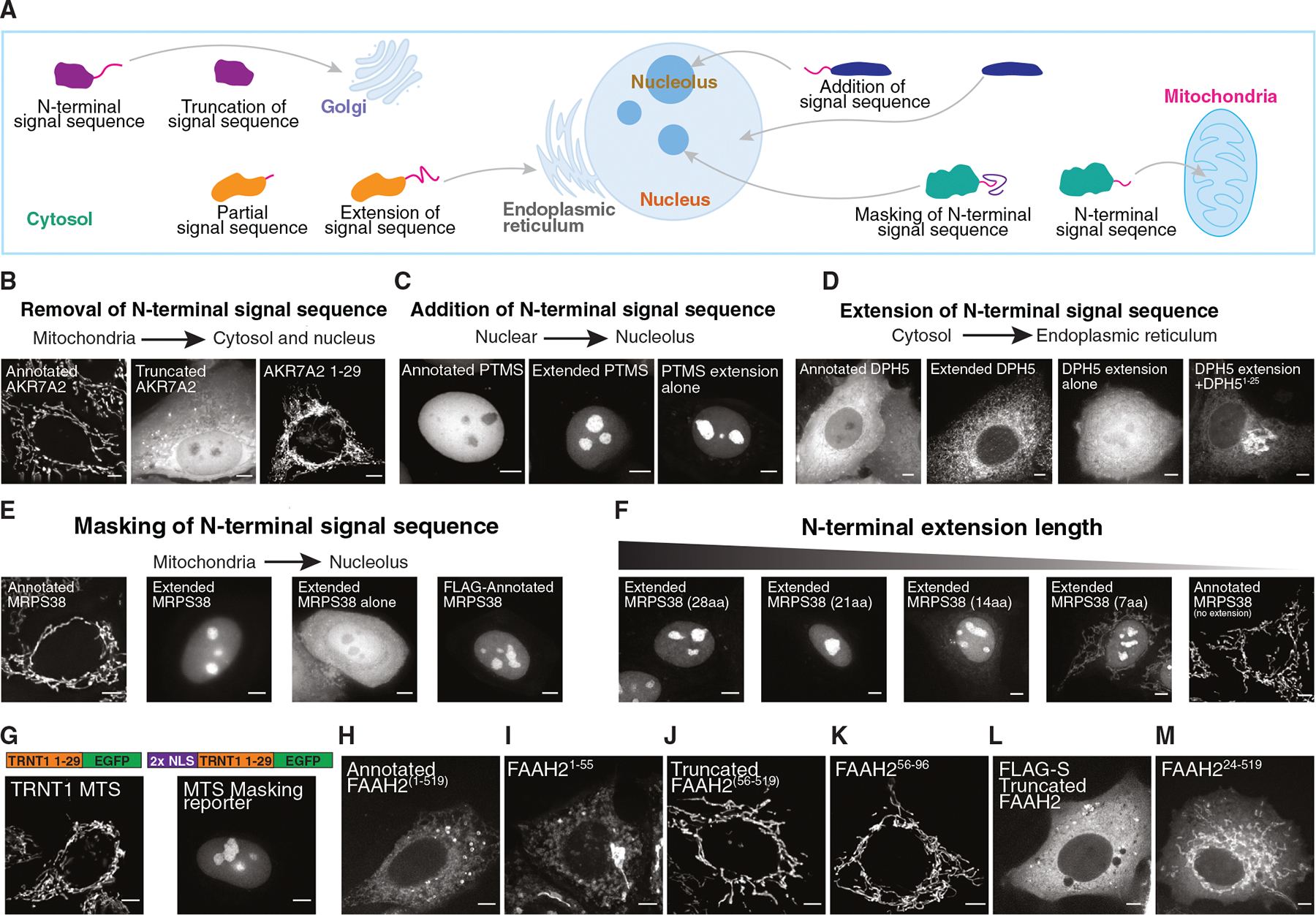
Mechanisms of differential localization and alternative start codon selection (A) Schematic model of the mechanisms by which alternative start codon selection may alter subcellular localization. (B) Left, live-cell imaging of annotated AKR7A2-GFP shows localization to mitochondria. Middle, live-cell imaging of truncated AKR7A2-GFP shows localization to cytosol and nucleus. Right, live-cell imaging of AKR7A21^−29^-GFP shows localization to mitochondria. (C) Left, live-cell imaging of annotated PTMS-GFP shows localization to the nucleus. Middle, live-cell imaging of extended PTMS-GFP shows localization to the nucleolus. Bottom, live-cell imaging of PTMS N-terminal extension alone tagged with GFP shows localization to the nucleolus. (D) Live-cell imaging of annotated DPH5-GFP (cytosol), extended DPH5-GFP (ER), the DPH5 extended region alone tagged with GFP (cytosol), and the DPH5 N-terminal extension with the first 25 amino acids of annotated DPH5 tagged with GFP (ER/Golgi). (E) Live-cell imaging of annotated AURKAIP1/MRPS38-GFP (mitochondrial), extended MRPS38/AURKAIP1-GFP (nucleolar), AURKAIP1/MRPS38 extended region alone tagged with GFP (diffuse), and N-terminally FLAG-tagged annotated MRPS38 with a C-terminal GFP (nucleolar). (F) Live-cell imaging of AURKAIP1/MRPS38-GFP with the indicated length of N-terminal extension. (G) Live-cell imaging of TRNT1 MTS-GFP without (left) and with (right) a 2× N-terminal nuclear localization signal to test generalizability of N-terminal masking. (H) Live-cell imaging of annotated FAAH2 with a C-terminal GFP (ER/lipid bodies). (I) Live-cell imaging of FAAH2 N terminus (1–55) with a C-terminal GFP (ER/lipid bodies/Golgi). (J) Live-cell imaging of truncated FAAH2 with a C-terminal GFP (mitochondrial). (K) Live-cell imaging of the N terminus of truncated FAAH2 (56–96) with a C-terminal GFP (mitochondrial). (L) Live-cell imaging of truncated FAAH2 with an N-terminal FLAG-S tag and a C-terminal GFP (cytosolic). (M) Live-cell imaging of FAAH2 24–519 with a C-terminal GFP (mitochondrial/cytosolic). Scale bar, 5 μm.

We next sought to define the molecular basis by which the subcellular localization of these protein variants is altered. We identified four strategies that create changes in subcellular localization (Figure 2A). First, N-terminal truncations can remove targeting signals, redirecting protein localization—as seen for aldo-keto reductase family 7 member A2 (AKR7A2), TRNT1, PNPO, glutathione-disulfide reductase (GSR), and cytidine/uridine monophosphate kinase 1 (CMPK1) (Figures 2B and S3A). Second, N-terminal extensions can introduce a complete signal sequence, such as the addition of a nucleolar signal to parathymosin (PTMS) or a mitochondrial targeting sequence to riboflavin kinase (RFK) (Figures 2C and S3B–S3D). Third, N-terminal extensions can complete partial signal sequences in the annotated protein. For example, the extended isoform of diphthamide biosynthesis 5 (DPH5) localizes to the endoplasmic reticulum (ER), whereas the annotated isoform remains in the cytosol (Figures 2D and S3E). However, the extended protein sequence alone is not sufficient to serve as an ER signal sequence. Instead, a functional ER signal sequence is only formed when the N-terminal extension is combined with the first 25 amino acids of DPH5 (Figures 2D and S3E), suggesting that the N-terminal extension completes the partial signal sequence in annotated DPH5.

In addition to these strategies, we identified an unanticipated fourth mechanism by which N-terminal extensions alter protein subcellular localization by “masking” or “internalizing” the annotated N-terminal targeting signal, thereby preventing it from being recognized. For example, the annotated isoform of AURKAIP1/MRPS38 localizes to mitochondria via its N-terminal targeting signal (Figures 2E and S3F). However, the N-terminally extended AURKAIP1/MRPS38 isoform localizes to the nucleus/nucleolus, likely because the extension displaces the mitochondrial targeting signal away from the N terminus, disrupting its function (Figure 2E). Indeed, we found that adding an unrelated N-terminal sequence (FLAG-S tag) to the annotated AURKAIP1/MRPS38 isoform also redirected it to the nucleus/nucleolus (Figure 2E). Similarly, the N-terminally extended DNA topoisomerase III alpha (TOP3A) isoform localizes to the nucleus instead of mitochondria through the internalization of its mitochondrial targeting sequences (Figure S3G).

To understand the molecular mechanism underlying N-terminal masking, we analyzed the minimal sequence length required to effectively conceal a mitochondrial targeting signal (Figure 2F). By analyzing the localization of partial N-terminally extended AURKAIP1/MRPS38-GFP constructs, we found that even a 7 amino acid N-terminal extension was sufficient for partial signal masking, although longer lengths were required for complete inhibition of mitochondrial targeting (Figure 2F). In addition, we tested whether ectopic N-terminal masking could suppress mitochondrial signals not normally regulated in this manner. Indeed, the addition of an N-terminal nuclear localization signal effectively masked the TRNT1 mitochondrial targeting signal (Figure 2G), supporting the idea that N-terminal masking may represent a generalizable regulatory mechanism. Our results suggest that even minor N-terminal extensions can function to mask N-terminal signal sequences, creating the potential for cryptic translational extensions to create alternatively localized protein products.

Finally, we also identified examples of alternative protein isoforms that integrate multiple of these four localization rules to create dual-localized proteins. For instance, the annotated fatty acid amide hydrolase 2 (FAAH2) isoform localizes to the ER and lipid droplets, with its N terminus (amino acids 1–55) serving as a transmembrane domain and localization signal (Figures 2H, 2I, and S3H). By contrast, the truncated FAAH2 isoform is targeted to mitochondria (Figure 2J) via an N-terminal mitochondrial targeting signal (amino acids 56–96) (Figure 2K). Adding an N-terminal FLAG-S tag to the truncated isoform disrupts mitochondrial localization, resulting in cytosolic localization (Figure 2L). This N-terminal FLAG tag on truncated FAAH2 effectively masks the mitochondrial targeting signal but does not localize to ER/lipid droplets because it lacks the canonical transmembrane domain, resulting in cytosolic localization. Masking of the FAAH2 mitochondrial targeting signal is also length dependent (Figure 2M), similar to AURKAIP1/MRPS38 (Figures 2E and 2F). Thus, differential localization is driven by the interplay between signal masking and the structural features of the N-terminal region (amino acids 1–55). Overall, this work highlights the diversity of mechanisms by which alternative translational isoforms regulate protein localization to allow a single gene to generate multiple protein isoforms with distinct subcellular localizations.

### Early eukaryotic origins of alternative translation initiation and dual localization

**Figure 3.**
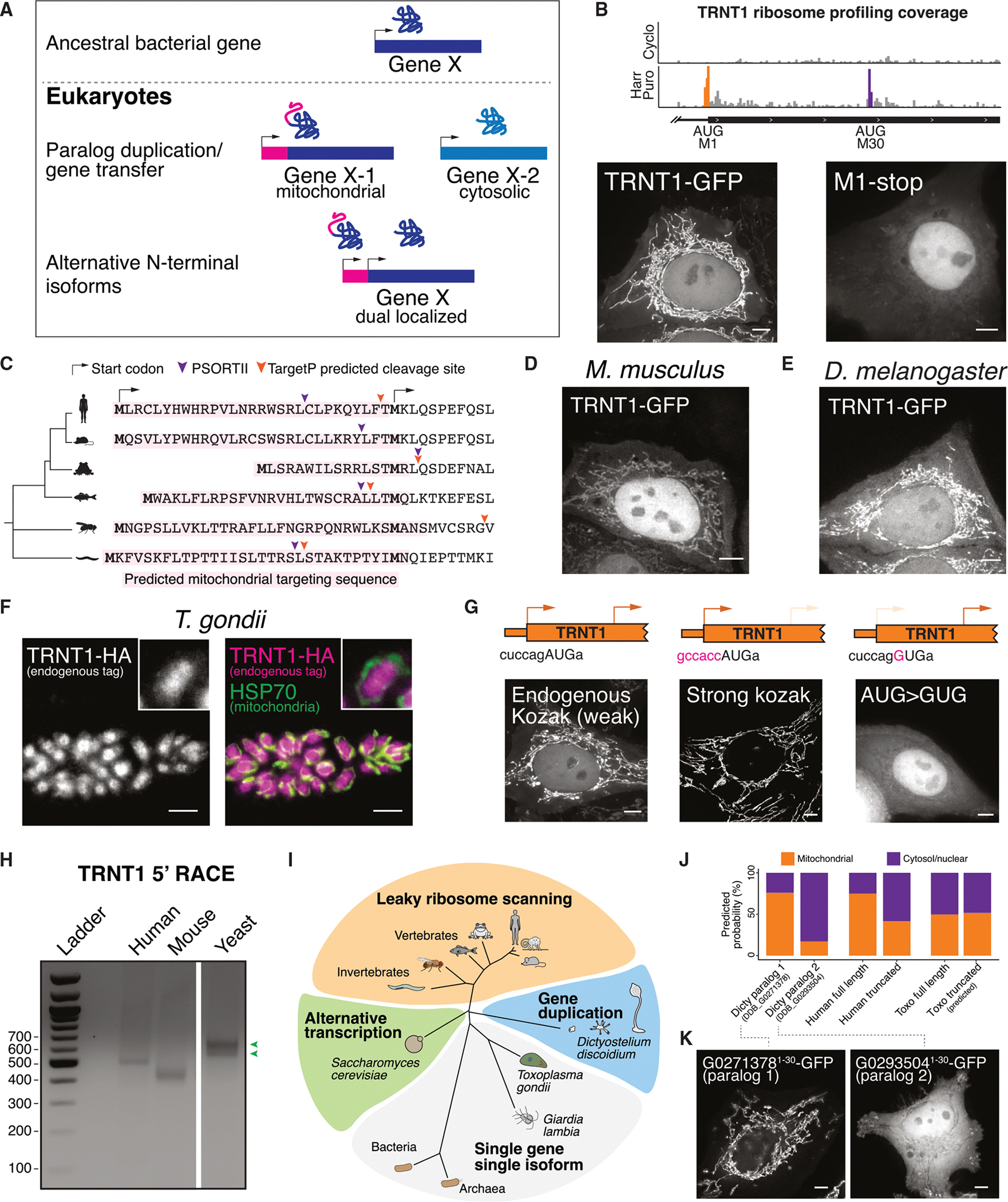
Early eukaryotic evolution of alternative TRNT1 start codons (A) Schematic of how alternative start codon selection generates dual-localized protein isoforms, which may replace paralog duplication/gene transfer during mitochondrial endosymbiosis. (B) Top, ribosome profiling reads around the TRNT1 start codons from HeLa cells. Orange bars represent ribosome-protected fragments at the annotated TRNT1 start codon, whereas purple reads represent reads at the alternative start codon. Bottom, live-cell imaging of cells expressing wild-type TRNT1 5′ UTR-coding sequence (CDS)-GFP (left) or TRNT1 5′ UTR-CDS-GFP but with a premature stop codon between Met1 and Met30 (right). (C) TRNT1 protein sequence alignment around the truncated start codon in Metazoans. The sequence with the magenta shade represents the predicted mitochondrial targeting signal from DeepLoc. The arrowheads indicate the predicted mitochondrial processing peptidase cleavage site by the indicated program. (D) Live-cell imaging of *M. musculus* TRNT1 5′ UTR-CDS-GFP in HeLa cells expressed from the safe harbor locus. (E) Live-cell imaging of *D. melanogaster* TRNT1 5′ UTR-CDS-GFP in HeLa cells expressed from the safe harbor locus. (F) Immunofluorescence imaging of endogenously tagged TRNT1-mCherry-HA in *T. gondii*, co-stained with the HSP70 mitochondrial marker. Inset region indicated by white box. (G) Live-cell imaging of human TRNT1 with the indicated start codon mutants. (H) 5′ rapid amplification of cDNA ends of TRNT1 from several organisms. The green arrowhead indicates different 5′ mRNA products for TRNT1 in *S. cerevisiae*. A white gap indicates image crop. (I) Cladogram showing the presence of TRNT1 alternative start codon selection and the underlying mechanism. (J) DeepLoc2.1 localization predictions for the two TRNT1 paralogs in *D. discoideum*, the alternative TRNT1 isoforms from humans, and *T. gondii* TRNT1 from the first and next downstream methionine. (K) Live-cell imaging of *D. discoideum* G0271378 and G0293504 N-terminal region tagged with C-terminal GFP, expressed in HeLa cells. Scale bar, 5 μm.

Among the alternative translational isoforms that displayed dual localization, the largest fraction involved changes in mitochondrial protein localization (Figures 1C–1E and S1G). The emergence of mitochondria was a pivotal event in eukaryotic evolution. As the mitochondrial genome became streamlined in the early eukaryotic ancestor, nuclear-encoded genes for DNA replication, protein translation, protein folding, and antioxidant activities needed to facilitate critical functions in both the mitochondria and other compartments within the cell.^7–10^ Our results suggest that one way in which cells enable these critical dual-localized protein functions in both the mitochondria and throughout the cell is by the generation of alternate protein isoforms. If this is the case, we reasoned that the existence of these alternate isoforms would be broadly conserved across eukaryotes, arising after mitochondrial acquisition and the streamlining of the mitochondrial genome (Figure 3A).

By analyzing evolutionary conservation (phylogenic *p* values [PhyloP])^41^ across vertebrates, we found that the alternative start codons for truncated isoforms are highly conserved for the tested dual-localized mitochondrial genes (Figure S4A), suggesting an evolutionary pressure to retain these dual-localized isoforms. For example, we identified conserved alternative translation initiation sites in GARS1 in human HeLa cells, mouse embryonic stem cells,^24^ and budding yeast^42^ that produce dual mitochondrial and cytosolic isoforms (Figures S4B and S4C). The mechanism that drives the production of alternative GARS1 isoforms is evolutionarily conserved from humans to budding yeast,^42^ occurring through leaky ribosome scanning due to a weak start codon at the Met1 initiation site and downstream translation initiation (Figure S4B). Similarly, we observed a strong conservation of alternative isoforms for TRNT1, which facilitates the maturation of both mitochondrial and nuclear-encoded tRNAs by adding a CCA sequence to their 3′ ends^43^ (Figures 3B, 3C, and S4D). To perform this dual function in distinct cellular compartments, we found that human TRNT1 undergoes alternative translation initiation at two sites: initiation at Met1 (AUG) that produces a mitochondrial-localized isoform, and initiation at methionine 30 (Met30) (AUG) that generates a nuclear/cytosolic isoform (Figure 3B). This mechanism is widely conserved across species (Figure 3C), including mice (Figure 3D), flies (Figure 3E), and budding yeast.^44^ These results suggest that there is a strong conservation of these alternative translational isoforms across eukaryotes.

Alternatively, we reasoned that eukaryotes that have dispensed with aspects of mitochondrial function would not have a selective pressure to retain this dual localization behavior. For example, *Trypanosoma brucei*^45^ and the apicomplexan parasite *Toxoplasma gondii*^46^ have functional mitochondria but lack mitochondrial-encoded tRNAs and instead import processed nuclear-encoded tRNAs into the mitochondria. In addition, the primitive eukaryote *Giardia lambia* lacks mitochondria altogether.^47^ Consistent with the absence of a requirement for mitochondrially localized TRNT1 in these species, we found that the alternative TRNT1 initiation site is absent in *G. lambia* and *T. brucei* (Figures S4E and S4F), suggesting the production of a single nuclear-localized TRNT1 translational isoform. To test this model directly, we monitored the subcellular localization of *T. gondii* TRNT1 (TGGT1_315810). We found that TRNT1 endogenously tagged with a C-terminal mCherry-hemagglutinin (HA) localized to the nucleus but not to mitochondria in *T. gondii* (Figure 3F), aligning with prior *T. gondii* mitochondrial proteomics data.^48^ These observations suggest that the alternative translation initiation sites in TRNT1 evolved from its ancestral bacterial/archaeal enzyme in early eukaryotes, likely after mitochondrial acquisition, but are only preserved in organisms that have retained mitochondrially encoded tRNAs. These results highlight the importance of alternative translation in generating dual-localized protein variants for mitochondrial function and eukaryotic evolution (Figure 3A).

### Diverse evolutionary pathways for production of dual-functional proteins

Our results indicate that a subset of dual-localized mitochondrial/cytosolic isoforms is deeply conserved, coinciding with mitochondrial endosymbiosis. We next sought to evaluate the gene regulatory strategies that control dual protein production across species. One major pathway of alternative start codon selection is through leaky ribosome scanning that allows for the usage of multiple start sites in a single mRNA.^38^ In vertebrates, TRNT1 is transcribed primarily as a single mRNA transcript (Figures S4G and S4H). TRNT1 alternative isoform translation occurs via leaky ribosome scanning due to a weak Kozak context at Met1 (Figures 3G and S4D) such that strengthening Met1 context favors the mitochondrial isoform (Figure 3G), whereas mutating Met1 (AUG>GUG) produces only the cytosolic/nuclear isoform (Figure 3G).

The decoding of a single gene into multiple protein products can also occur through alternative transcription initiation.^49–51^ In this case, the use of distinct promoter sites can result in mRNAs of differing lengths such that the shorter product lacks the ability to use the upstream translation start site. Indeed, in budding yeast (*S. cerevisiae*),^44^ the TRNT1 homolog CCA1 produces two distinctly localized isoforms through alternative transcription initiation rather than leaky scanning (Figures 3H, 3I, and S4I).

Lastly, instead of encoding a dual-localized TRNT1 as a single gene, the slime mold *Dictyostelium discoideum* has two TRNT1 paralogs with distinct predicted localizations. One paralog (DDB_G0271378) contains a strong N-terminal mitochondrial targeting signal (MTS) and is predicted to localize to the mitochondria, whereas the other (DDB_G0293504) lacks an N-terminal MTS and is predicted to localize to the nucleus (Figures 3I, 3J, S4J, and S4K). Indeed, based on ectopic expression in human cells, we found that the N terminus of DDB_G0271378 functions as a mitochondrial targeting signal, whereas the DDB_G0293504 paralog did not specify subcellular localization (Figure 3K). These findings highlight the diverse gene regulatory strategies that act across evolution to maintain the production of dual-localized activities for a single enzyme to create both mitochondrial and nuclear populations (Figure 3I).

### Generation of isoform-selective knockout alleles reveals isoform-specific functions

We next sought to identify and analyze the downstream functions of differentially localized alternative N-terminal isoforms. We focused on AURKAIP1/MRPS38, where a 28-amino-acid N-terminal extension from a CUG start codon redirects the protein to the nucleolus by masking its mitochondrial targeting signal (Figures 2E and S5A). AURKAIP1/MRPS38 has an established mitochondrial ribosome subunit^52^ but has no known nucleolar role. Therefore, we first confirmed expression of the extended isoform using mass spectrometry, immunofluorescence, and luciferase assays (Figures S5B–S5D). To investigate the mechanism underlying the usage of this upstream CUG start codon in AURKAIP1/MRPS38, we analyzed the 5′ mRNA sequence. We identified a strong RNA secondary structure downstream of the CUG translational extension start site in AURKAIP1/MRPS38, which may act to stall scanning ribosomes to promote CUG initiation (Figure S5E).^16,53–56^ Indeed, disrupting this RNA secondary structure reduced CUG-driven translation and eliminated the nucleolar isoform, emphasizing its functional importance (Figures S5F and S5G).

**Figure 4.**
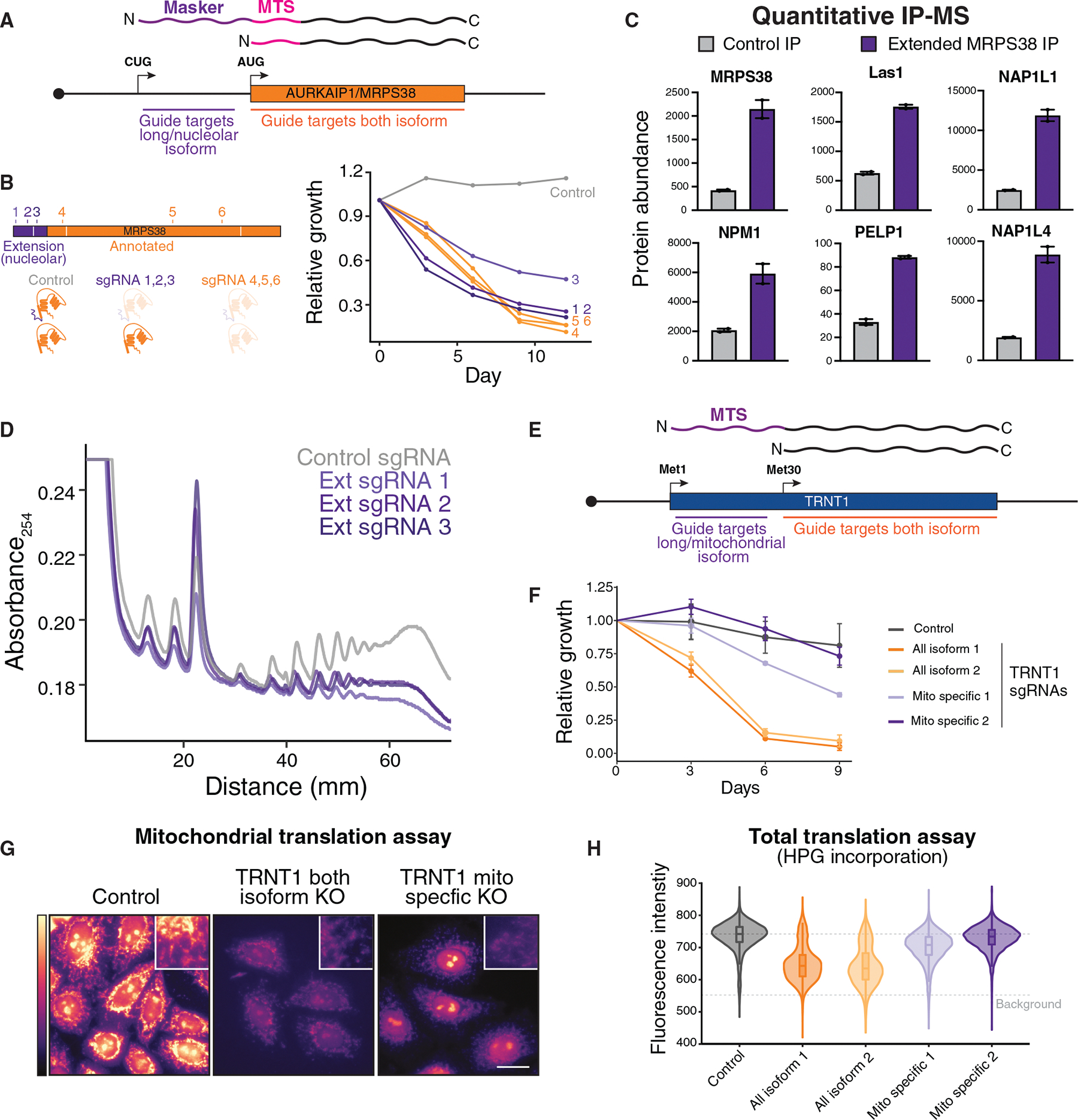
Alternative start codon selection promotes dual protein function (A) Schematic of the CRISPR-Cas9 system to generate polyclonal isoform-specific AURKAIP1/MRPS38 knockout. Indels after the CUG start codon but before the AUG start codon will cause frameshift alleles to disrupt extended AURKAIP1/MRPS38. (B) Schematic of gRNAs to selectively deplete MRPS38 isoforms for competitive growth assays (left). Line plot showing the relative growth of knockout cells (right). Guide RNAs (1–3) specifically eliminating the CUG-initiation MRPS38 extension result in a growth defect. (C) Quantitative MS analysis of extended MRPS38/AURKAIP1 immunoprecipitation reveals interactions with several nucleolar and nuclear proteins. (D) Sucrose gradient profiles from day 6 control and MRPS38 extension knockout cells. (E) Schematic for CRISPR-Cas9 system to generate isoform-specific knockouts for genes with N-terminal truncations. (F) Competitive growth assay results in cells expressing various gRNAs to selectively deplete TRNT1 isoforms. (G) Imaging of mitochondrial translation rate assay in control and isoform-specific TRNT1 knockouts. The inset region, representing a zoom in on mitochondrial stain, is indicated by a white box. Scale bar, 25 μm. (H) Violin plot showing flow cytometry results from total HPG incorporation assay in control and isoform-specific TRNT1 knockouts. The dotted line represents median HPG intensity for control knockout cells (top dashed line) and cells without HPG (bottom dashed line, background defined by a no HPG control).

To investigate the function of the extended AURKAIP1/MRPS38 isoform, we generated isoform-selective knockout alleles using a CRISPR-Cas9-based strategy (Figure 4A). We designed guide RNAs targeting the region between the extended and annotated start codons, inducing frameshift mutations that selectively disrupt the extended isoform while preserving the annotated form. By contrast, targeting regions downstream of the annotated start codon is predicted to disrupt all AURKAIP1/MRPS38 isoforms. Knocking out all AURKAIP1/MRPS38 isoforms resulted in substantial growth defects, consistent with its known role as a core mitochondrial ribosome subunit (Figure 4B, orange lines). In addition, selectively eliminating the extended isoform also impaired cell growth (Figure 4B, purple lines), suggesting an independent function.

We next investigated the function of extended nucleolar AURKAIP1/MRPS38. Using immunoprecipitation-mass spectrometry (IP-MS), we found that the extended AURKAIP1/MRPS38 isoform interacts with multiple nucleolar ribosome biogenesis factors—including LAS1-like ribosome biogenesis factor (LAS1); nucleophosmin 1 (NPM1); and proline-, glutamate-, and leucine-rich protein 1 (PELP1)—as well as nuclear proteins such as nucleosome assembly protein 1-like 1 NAP1L1 and nucleosome assembly protein 1-like 4 NAP1L4 (Figures 4C and S5H; Table S3). Given its interaction profile and nucleolar localization, we tested whether this extended isoform contributes to cytosolic translation. Indeed, selectively eliminating the extended AURKAIP1/MRPS38 isoform led to a global reduction in cytosolic translation, as assessed by polysome profiling (Figure 4D). However, extended AURKAIP1/MRPS38 knockout cells exhibited normal rates of rRNA transcription (Figure S5I), suggesting that the extended isoform functions downstream of or independently from rRNA synthesis to promote cytosolic translation. These results suggest that AURKAIP1/MRPS38 produces two functionally distinct isoforms: the annotated isoform supporting mitochondrial translation and the extended isoform promoting cytosolic translation (Figure S5J). Consistent with this, recent genome-wide perturb-seq data show that AURKAIP1/MRPS38 displays functional clustering with cytosolic ribosome genes in RPE1 cells,^57^ indicative of similar cellular phenotypes. Thus, alternative translation can generate compartment-specific isoforms that together fulfill essential gene functions.

We also identified multiple other genes that are predicted to rely on alternative N-terminal isoforms to fulfill their established functions. For instance, TRNT1 has documented roles both within and outside the mitochondria.^43^ However, the annotated isoform is exclusively mitochondrial, suggesting that the proposed nuclear/cytosolic functions may instead be mediated by the N-terminal truncated isoform (Figure 3). To test this, we used CRISPR-Cas9 to generate isoform-selective TRNT1 knockouts (Figure 4E). We found that guide RNAs targeting both TRNT1 isoforms conferred greater fitness defects than those disrupting only a single isoform (Figure 4F). Based on L-homopropargylglycine (HPG) incorporation to monitor both mitochondrial^58^ and total translation,^59^ we found that guide RNAs eliminating both TRNT1 isoforms reduced both global translation and mitochondrial translation, whereas guide RNAs targeting the longer TRNT1 isoform only eliminated mitochondrial translation (Figures 4G and 4H). Overall, these results highlight the importance of alternative start codon selection in generating multiple protein isoforms, each of which contributes to cellular function and viability.

### ISAs in rare human diseases

**Figure 5.**
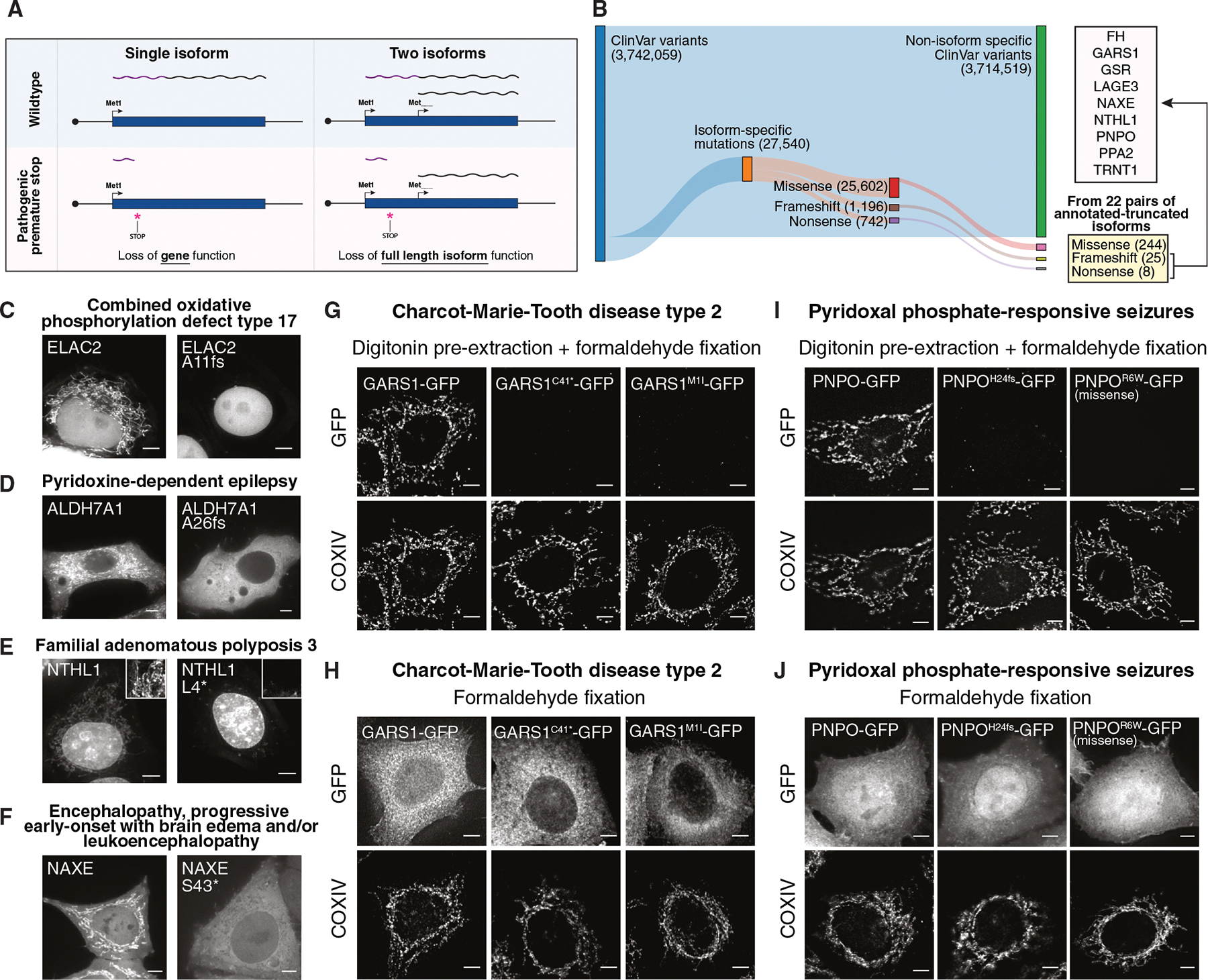
Rare disease mutations target specific alternative translational isoforms (A) Schematic model showing how a pathogenic premature stop codon mutation would affect a gene with one (left) or multiple (right) translation initiation sites. With multiple alternative start codons, the premature stop codon mutation might not eliminate all protein variants from the gene. Similarly, frameshift or missense mutations may selectively impact a single isoform of a gene. (B) Sankey plot summary of isoform-specific mutations from the ClinVar database. From the 22 pairs of differentially localized annotated-truncated isoforms, 11 of these pairs had isoform-specific mutations that would eliminate the protein product from the first start codon. (C) Live-cell imaging of wild type of pathogenic A11fs ELAC2 5′ 5′ UTR-CDS-GFP. We note that ELAC2 was not a part of our initial screen and was tested based on Rossmanith^18^ and this allele. (D) Live-cell imaging of wild type of pathogenic S26fs ALDH7A1 5′ 5′ UTR-CDS-GFP. ALDH7A1 was not included in the initial localization screen but was subsequently tested based on this ClinVar allele. (E) Live-cell imaging of wild type of pathogenic L4* NTHL1 5′ 5′ UTR-CDS-GFP. The inset region zooming in on mitochondrial localization is indicated by a white box. Inset images are scaled to be brighter than the full-size image. (F) Live-cell imaging of wild type of pathogenic S43* NAXE 5′ 5′ UTR-CDS-GFP. (G) Immunofluorescence of GARS1 5′ UTR-CDS-GFP with different ClinVar mutations, co-stained with COXIV (mitochondrial marker) in cytosolic pre-extracted cells. (H) Same as (G) but without pre-extraction. Wild-type GARS1 localizes to the cytosol, nucleus, and mitochondria, whereas the C41* and M1I pathogenic variants eliminate the production of mitochondrial GARS1, initiating at Met1. (I) Immunofluorescence of PNPO 5′ UTR-CDS-GFP with different ClinVar mutations, co-stained with COXIV (mitochondrial marker) in cytosolic pre-extracted cells. R6W is an isoform-specific missense allele that is predicted to disrupt mitochondrial localization. (J) Same as (J) except without pre-extraction. Scale bar, 5 μm.

Our analysis highlights the essential role of alternative protein isoforms that enable dual activities both within and outside the mitochondria. Given the importance of mitochondrial function for human health, we next assessed whether there are patient mutations that affect N-terminal isoforms in rare genetic diseases (Figure 5A). To identify pathogenic mutations that target a specific alternative translational isoform for a gene, we analyzed the ClinVar database.^60^ Of the 3,334,918 ClinVar variants examined, we identified 24,183 isoform-specific mutations associated with rare diseases. This includes 22,487 missense, 660 nonsense, and 1,036 frameshift mutations (Figures 5B and S6A), with 258 genes (24%) containing a nonsense or frameshift mutation that is predicted to eliminate the annotated but not truncated isoform of a gene (Table S4). Of the 22 pairs of annotated and N-terminal truncated isoforms analyzed in the localization screen (Figure 1E; Table S2), 9 genes (fumarate hydratase [FH], inorganic pyrophosphatase 2 [PPA2], GARS1, GSR, L antigen family member 3 [LAGE3], NAD[P]HX epimerase [NAXE], nth-like DNA glycosylase 1 [NTHL1], PNPO, and TRNT1) contain a nonsense or frameshift mutation between the annotated and truncated start codons that is associated with a rare disease (Table S4).

To test the impact of these disease mutations, we evaluated specific genes. We identified mutations in elaC ribonuclease Z 2 (ELAC2^18^) (c.30_36del; p.Ala11fs) in combined oxidative phosphorylation defect type 17 disease (Figure 5C), aldehyde dehydrogenase 7 family member A1 (ALDH7A1) (c.76_82del; p. Ala26fs) in pyridoxine-dependent epilepsy (Figure 5D), NTHL1 (c.11T>A; p.Leu4Ter) in familial adenomatous polyposis 3 (Figure 5E), NAXE (c.128C>A; p.Ser43Ter) in early-onset progressive encephalopathy characterized by brain edema and/or leukoencephalopathy (Figure 5F), GARS1 (c.123C>A; p.Cys41Ter) in Charcot-Marie-Tooth disease type 2 (Figures 5G and 5H), and PNPO (c.69dup; p.His24fs) in patients with pyridoxal phosphate-responsive seizures (Figures 5I and 5J), among others (Figures 5B and S6B–S6E; Table S4). These premature stop codons and frameshift mutations would typically be interpreted as complete loss-of-function alleles when relying solely on annotated gene models (Figure 5A). However, the presence of downstream alternative translation initiation sites for these genes instead predicts that these mutations would only eliminate the longer protein isoform, leaving the alternative N-terminally truncated protein isoform intact (Figure 5A). Indeed, we found that these disease alleles selectively abolished the expression of the longer mitochondrial isoforms initiated from the first AUG while preserving the cytosolic/nuclear-localized truncated isoform (Figures 5C–5J). Consistent with these alleles being partial rather than complete loss of gene function, the patient with a mitochondrial isoform-specific NAXE mutation (c.128C>A; p.Ser43Ter; Figure 5F) developed disease symptoms relatively late—at age 12—and lived until 26 (Leiden Open Variation Database [LOVD] ID: 00334932). This contrasts with individuals carrying mutations that disrupt both isoforms, who typically present in infancy and pass away within the first few years of life.^61^

In addition to isoform-selective nonsense and frameshift alleles, we analyzed isoform-selective missense alleles. To identify isoform-specific missense alleles that specifically alter the localization of one isoform, we used DeepLoc2.1 to predict changes in subcellular localization for the wild-type and isoform-specific missense allele. We analyzed 25,602 isoform-specific missense alleles across 931 genes and found 250 missense mutations that were predicted to alter the localization of one isoform (Figure S6F; Table S4). For example, we found that PNPO R6W (c.16C>T), which is a variant of unknown significance associated with pyridoxal phosphate-responsive seizures, weakens the mitochondrial localization signal of the annotated isoform but does not affect the function or activity of the downstream translation product (Figures 5I and 5J).

These results highlight the significance of alternative start codon usage and N-terminal isoforms in the context of rare diseases, emphasizing the need to consider the isoform-specific impact of mutations in genetic and clinical studies. To facilitate the identification and analysis of isoform-specific mutations for newly generated patient genetic sequencing data, we developed SwissIsoform (https://github.com/cheeseman-lab/swissisoform)—a platform designed to determine whether a mutation selectively affects one or more protein isoforms for genes with a translational truncation (Figure S6G).

### Isoform-selective frameshift alleles in TRNT1 can result in unique clinical symptoms

**Figure 6.**
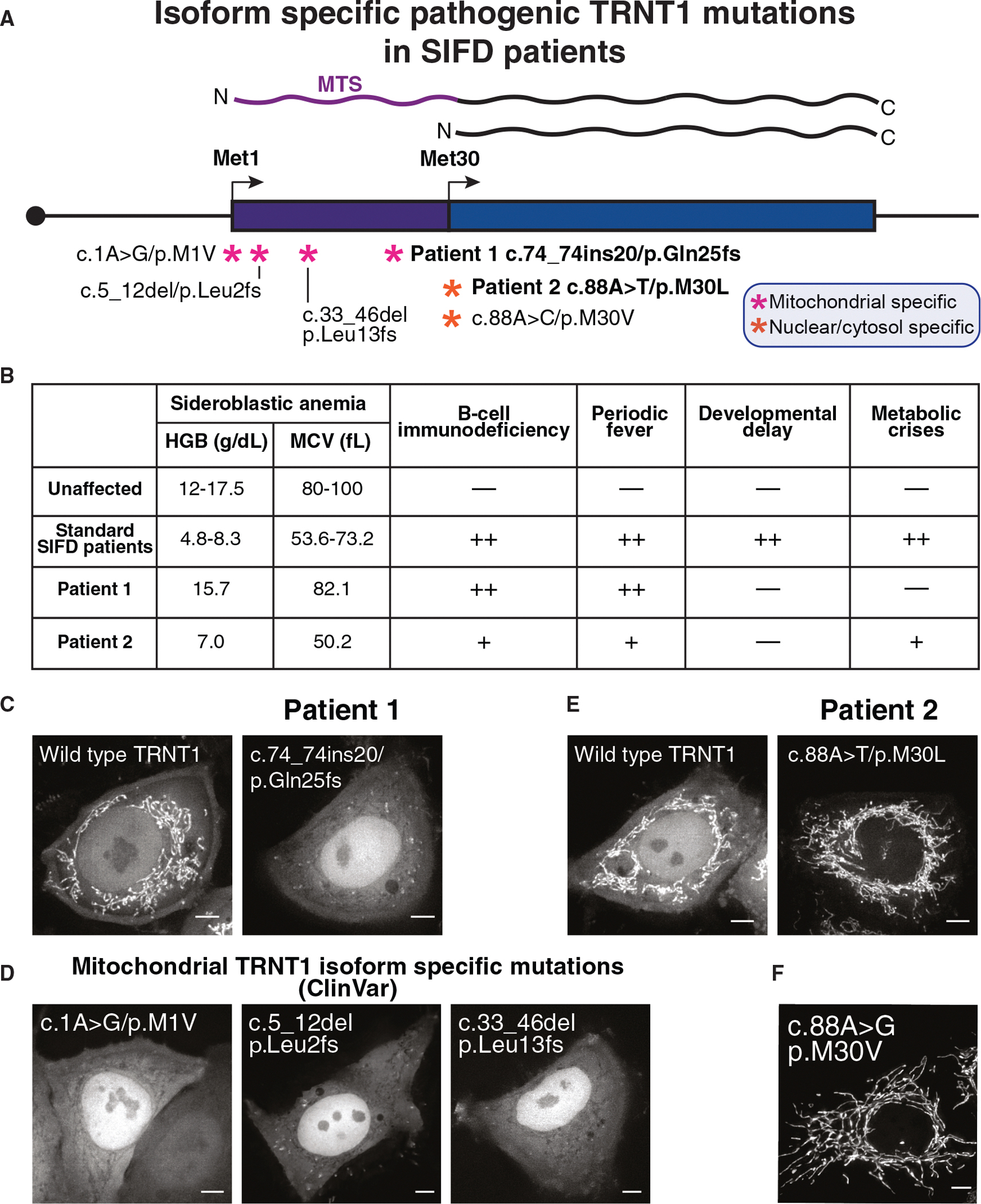
TRNT1 isoform-selective mutations in atypical SIFD patients (A) Schematic representation of isoform-specific TRNT1 SIFD mutations from ClinVar and patients from Boston Children’s Hospital. (B) Table showing the severity of clinical symptoms from SIFD patients. HGB and MCV measurements for standard SIFD patients from Wiseman et al.^64^ and unaffected individuals from El Brihi et al.^65^ HGB, hemoglobin levels; MCV. Metabolic crises include nausea, vomiting, diarrhea, and abdominal pain. (C) Live-cell imaging of wild-type and patient 1 TRNT1 isoform-specific mutation. (D) Live-cell imaging of the selected mitochondrial isoform-selective TRNT1 alleles. (E) Live-cell imaging of wild-type and patient 2 TRNT1 isoform-specific mutation. (F) Live-cell imaging of TRNT1 M30V. Scale bar, 5 μm.

We next sought to leverage the understanding of dual-localized isoforms to evaluate whether isoform-specific mutations can improve our understanding of rare disease pathologies. In patients with sideroblastic anemia, B cell immunodeficiency, periodic fevers, and developmental delays (SIFD), mutations downstream of Met30—including premature stop codons, frameshifts, and catalytic region mutations—are predicted to disrupt both the mitochondrial and nuclear TRNT1 isoforms (Figure S7A).^62,63^ By contrast, our model predicts that nonsense or frameshift mutations upstream of the Met30 start codon would specifically impact the longer TRNT1 isoform. Interestingly, among a cohort of Boston Children’s Hospital SIFD patients, we identified two patients with unique SIFD phenotypes that carry mutations predicted to selectively impact specific TRNT1 isoforms (Figures 6A and 6B), as described below.

Patient 1 is a developmentally normal 58-year-old male with a lifelong history of periodic fevers treated since childhood with colchicine and/or tumor necrosis factor alpha (TNF-α) inhibitors for a presumptive diagnosis of familial Mediterranean fever (FMF). This patient has received 5 transfusions during his lifetime in the context of recurrent pulmonary and gastrointestinal hemorrhage. However, in contrast to classic SIFD patients, he is not chronically anemic. The patient was diagnosed with chronic myeloid leukemia at age 42 and treated into molecular remission with dasatinib and subsequently developed total color and night blindness. In recent years, he was noted to be hypogammaglobulinemic, and the fevers have accelerated in frequency and are associated with multiple episodes of culture-negative bronchitis and a pustular rash. Molecular analysis for periodic fevers at age 50 revealed TRNT1 mutations (NM_182916.3, c.74_74ins20/c.1056+11G; p.Gln25His*6/p?) confirmed to be biallelic by segregation analysis in his parents and a brother (Figure S7B). Patient 1 is not anemic (hemoglobin levels [HGB] 15.7 g/dL) but is slightly microcytic (mean corpus volume [MCV] 82.1 fL) and has not had a bone marrow examination (Figure 6B). These clinical features are in contrast to the classic SIFD patients with mutations targeting both isoforms of TRNT1, who are severely anemic.^64^

Annotated gene models predict the p.Gln25His*6 mutation would be a null allele (Figure 5A). By contrast, as TRNT1 uses an alternative start codon at Met30 to produce a nuclear isoform, this downstream variant is not expected to be impacted by this mutation due to leaky ribosome scanning. Indeed, we found that expression of a TRNT1-GFP construct containing the patient frameshift mutation selectively eliminated the mitochondrial isoform but retained nuclear localization (Figure 6C). We also identified a patient from ClinVar (SCV005069659.1) containing a mitochondrial TRNT1 isoform-specific mutation (c.33_46del (p.Leu13fs)) who is reported to display retinal dystrophy without anemia similar to patient 1 (Figure 6D). These findings suggest that the unique SIFD clinical phenotypes in patient 1 may arise from the selective disruption of mitochondrial TRNT1, rather than disrupting all TRNT1 isoforms.

These findings highlight the importance of considering alternative start codons in interpreting patient mutations. Without accounting for alternative start codons in TRNT1 (Figures 6A–6D) or NAXE (Figure 5F), these frameshift or nonsense alleles would be misinterpreted as complete loss-of-function mutations. Furthermore, our results highlight how isoform-specific alleles can result in distinct clinical symptoms compared with alleles affecting all isoforms of the gene and underscore the importance of considering alternative start codon context when evaluating variant pathogenicity and diagnosis.

### Pathogenic alternative start codon alleles can lead to isoform-selective disease phenotypes

Genetic changes downstream of an annotated start codon that do not result in premature termination are typically classified as missense alleles and assessed based on the specific amino acid change. However, mutations that impact a downstream alternative start codon must also be interpreted at the level of translation. In our analysis of the Boston Children’s Hospital cohort, we also identified a second patient with isoform-specific TRNT1 mutations and distinct SIFD symptoms (Figure 6B). Patient 2 is a 10-year-old developmentally normal Haitian/African American child who presented at age 16 months with microcytic anemia (HGB 7.0 g/dL, MCV 50.2 fL) and oral candidiasis, mild B cell and CD8^+^ T cell lymphopenia without hypogammaglobulinemia (Figure 6B). Bone marrow examination showed numerous ringed sideroblasts. Ophthalmologic examination showed mild retinal dystrophy. His subsequent clinical course has been characterized by a stable anemia and 2–3 febrile episodes associated with abdominal pain, nausea, and vomiting per year that typically respond to hydration alone.

Genetic testing revealed biallelic TRNT1 mutations (c.88A>T/c.461C>T; p.Met30Leu/p.Thr154Ile) (Figure S7C). Of these mutations identified in patient 2, Thr154Ile occurs in the catalytic region and is predicted to reduce the function of both nuclear and mitochondrial TRNT1 isoforms (AlphaMissense score = 0.989).^66^ By contrast, the M30L mutation would be assessed as a conserved hydrophobic amino acid change when considering only the longer TRNT1 isoform. However, based on our data, this change is predicted to impact the alternative downstream start codon mutation in TRNT1 and disrupt translation initiation at the alternative TRNT1 start codon, reducing the nuclear isoform. Consistent with this, C-terminal GFP-tagged fusion assays showed a strong depletion of nuclear TRNT1, whereas the mitochondrial isoform remained largely unaffected (Figure 6E). In addition to patient 2, a previously reported Chinese patient carrying a c.88A>G/c.363G >T allele (p.Met30Val/p.Glu121Asp) also had a similarly mild phenotype characterized by normal neurocognitive development, recurrent pulmonary infections, and autoimmune musculoskeletal complications.^67^ Modeling this TRNT1 M30V allele also disrupted the nuclear truncated TRNT1 isoform, leaving the mitochondrial TRNT1 isoform unaffected (Figure 6F). These results suggest the unique SIFD phenotype in patient 2 may arise from the loss of nuclear TRNT1 activity, with mitochondrial function preserved.

Standard predictive tools such as AlphaMissense classify M30L and M30V in TRNT1 as benign (scores of 0.278 and 0.135 out of 1.0, respectively). Consistently, ClinVar lists the TRNT1 M30V variant (SCV001220948.3) as a variant of uncertain significance, noting that “algorithms developed to predict the effect of missense changes on protein structure and function (SIFT, PolyPhen-2, and Align-GVGD) all suggest that this variant is likely to be tolerated.” This classification may lead clinicians and researchers to overlook the potential impact of this allele. However, our findings demonstrate that both M30L and M30V markedly impair translation of the nuclear TRNT1 isoform, supporting their pathogenicity.

To identify additional such alleles that impact downstream start sites, we analyzed the ClinVar database and found 467 “missense” variants across 332 genes that mutate an alternative AUG start codon (Figure S7D; Table S5). Among these, 54 variants affecting 42 genes are predicted to abolish production of a truncated N-terminal isoform that is expected to have distinct subcellular localization compared with the annotated full-length protein (Table S5), such as ALDH7A1 (c.85A>G/p.Met29Val; Figure 5D). These results underscore the critical need to account for alternative start codons when assessing the functional consequences of these genetic variants.

## DISCUSSION

The critical importance of the mitochondria is highlighted by the presence of mutations that compromise its activity in a wide range of rare human genetic diseases. Mitochondrial endosymbiosis required the ability to perform the same core biological activities both within the mitochondria and within the host cell. One key strategy for adapting to mitochondrial acquisition involves paralog duplication, followed by the evolution of a mitochondrial targeting sequence in one paralog to enable dual functionality in both compartments. Here, we demonstrate that alternative start codon selection provides an additional pervasive mechanism for generating dual-localized mitochondrial and nuclear/cytosolic protein isoforms, ensuring the maintenance of core cellular processes across these distinct locations (Figures 1 and 2). Furthermore, we provide evidence that alternative start codon usage has early eukaryotic origins, potentially arising from a bacterial/archae-bacterial ancestor soon after the emergence of mitochondria (Figure 3). Beyond dual-localized mitochondrial/cytosolic proteins, we also identify alternative protein variants that localize to other organelles, suggesting a broader role for alternative translation initiation in organelle-specific proteome diversification.

The use of alternative start codons within a single mRNA also has important implications for interpreting disease mutations (Figures 5 and 6). Rather than assuming a premature stop codon or frameshift mutation eliminates all protein function, we show that nonsense or frameshift mutations between two translation initiation sites selectively eliminate the longer protein isoform while having limited effects on downstream initiation (Figure 6). Notably, we identify isoform-specific TRNT1 mutations as key factors in the heterogeneous clinical phenotypes of SIFD (Figure 6). Beyond premature stop and frameshift mutations, isoform-selective missense mutations may also contribute to disease (Figures 5I and 5J). Mutations that alter the Kozak context, annotated start codons (Figures 5G and 6D), or alternative start sites (Figures 6E and 6F) could also lead to isoform-selective effects that alter the ratio of alternate N-terminal isoforms. Our results indicate that even conservative amino acid substitutions—such as the TRNT1 M30L variant—that are predicted to be structurally benign can be pathogenic if they disrupt alternative start codons (Figures 6E and 6F). These findings highlight the importance of considering alternative start sites when interpreting variant pathogenicity in American College of Medical Genetics and Genomics (ACMG) guidelines, ClinVar annotations, and clinical genetic testing. Furthermore, mutations affecting alternative promoters, RNA secondary structure, or other sequence elements may similarly be pathogenic by altering protein isoform ratios. Given the plethora of rare diseases with unexplored molecular mechanisms and variants of unknown significance,^68^ our findings underscore the need to systematically identify and analyze rare disease mutations that impact specific protein isoforms.

Based on the impact of the disease alleles analyzed here, we propose the term ISAs to describe these variants. ISAs can lead to distinct cellular consequences and clinical phenotypes compared with alleles that impact all isoforms of a gene. Their existence highlights the limitations of variant analyses that consider only annotated ORFs, potentially overlooking clinically relevant variants.

### Limitations of the study

Our work highlights a wide range of dual-localized alternate protein isoforms. However, our analysis may not fully capture the range of these. For our analysis, we focused particularly on non-nuclear organelle localization due in part to the fact that DeepLoc has limited accuracy in distinguishing nuclear from cytosolic localization.^69^ In addition, our localization-based studies analyzed the subcellular localization of dozens of N-terminal isoform pairs using C-terminal GFP fusions. The addition of a C-terminal GFP tag allows for the direct visualization of its localization; however, the presence of this tag may alter the localization of some isoforms. Despite this limitation, our experimental results align well with the computational predictions by DeepLoc2.1. Although our results provide strong evidence that alternative start codons give rise to isoforms with distinct N termini and differential localization, confirming their size differences by western blot proved challenging. This is largely due to the fact that mitochondrial targeting signals and ER secretion signals are cleaved upon import into these organelles, and the resulting cleavage products often resemble the size of the alternative isoform initiated from downstream start codons—causing both forms to migrate similarly on an SDS-PAGE gel. Finally, a major challenge in evaluating the functional impact of rare disease alleles lies in the limited number of available patient cases and the way in which these are reported in databases such as ClinVar. For example, the precise clinical features associated with the TRNT1 ISAs would benefit from the identification of additional patients carrying these variants.

## RESOURCE AVAILABILITY

### Lead contact

Requests for resources and reagents should be directed to and will be fulfilled by the lead contact, Iain Cheeseman (icheese@wi.mit.edu).

### Materials availability

All unique/stable reagents generated in this study are available from the lead contact with a completed materials transfer agreement.

### Data and code availability

- The mass spectrometry proteomics data have been deposited to the ProteomeXchange Consortium via the PRIDE^70^ partner repository with the dataset identifier PXD062112 and https://doi.org/10.6019/PXD062112. These data are publicly available as of the date of publication.
- Original microscopy and gel images have been deposited with Mendeley Data: https://doi.org/10.17632/m6rj76j5hm.1; https://doi.org/10.17632/nv9jb8f5mg.1; https://doi.org/10.17632/r3w7hr7j36.1 and are publicly available as of the date of publication.
- Code for SwissIsoform v1.0.0 is available at GitHub: https://github.com/cheeseman-lab/swissisoform and Zenodo: https://doi.org/10.5281/zenodo.17241986. The code is publicly available as of the date of publication.
- Any additional information required to reanalyze the data reported in this paper is available from the lead contact upon request.

## STAR★METHODS

### EXPERIMENTAL MODEL AND STUDY PARTICIPANT DETAILS

#### Tissue culture

HeLa and HEK293T cells were cultured in DMEM with 10% heat-inactivated fetal bovine serum, 2 mM L-glutamine and 100 U/mL penicillin-streptomycin at 37°C with 5% CO2.

#### Human patients and ethics approval

Human samples were obtained with written informed consent under a human subject’s research protocol (06–12-0536) approved by the institutional review board at Boston Children’s Hospital. Patient 1 with the isoform-selective frameshift TRNT1 allele is a 58-year-old male. Patient 2 with the TRNT1 M30L mutation is a 10-year-old male

### METHOD DETAILS

#### Molecular biology and cell line generation

All cDNA, except for non-human genes analyzed in this study, was amplified from HeLa cell cDNA. HeLa cell cDNA was generated via reverse transcription using the Maxima First Strand cDNA Synthesis Kit (K1641), following the manufacturer’s instructions. A summary of the protein sequences tested can be found in Table S2. Non-human genes were synthesized by Twist Bioscience.

To test whether the N-terminus contains sufficient signal sequences, we used the entire region upstream of the truncated start codon. For example, in the case of NAXE, where the N-terminal truncation occurs at Met52, we tested the region from Met1 to Val51 for a localization signal. For the analysis of N-terminal extensions, we included the entire extension, except in the case of DPH5. To determine whether extensions “mask” or “internalize” annotated N-terminal signal sequences, we appended a FLAG-S tag (MDYKDDDDKGKETAAAKFERQHMDSGGT) to the N-terminus of the annotated protein.

The amplified cDNA was inserted downstream of the TRE3G promoter and upstream of the MYC-TEV-EGFP tag using Gibson assembly. A SalI cut site was positioned between the TRE3G promoter and the cDNA construct. The plasmid also contained a puromycin resistance marker, a reverse tetracycline-controlled transactivator, and a C-terminally EGFP-tagged transgene, flanked by safe harbor AAVS donor homology arms. All transgenes were expressed from the safe harbor AAVS1 locus.^78^ Donor plasmid also contained the puromycin resistance marker, reverse tetracycline-controlled transactivator, the transgene, flanked by safe harbor AAVS donor homology arms. To generate dox-inducible GFP cell lines, 500 ng donor plasmid and 500 ng pX330-sgRNA AAVS1 were co-transfected using Lipofectamine 2000. 2 days post transfection, cells were selected with 0.4 μg/mL puromycin for at least 3 days.

When optimizing the Kozak sequence, the 6 nucleotides upstream of the start codon was mutated to GCCACC. The +4 position was kept as the endogenous sequence to not change the protein coding sequence.^79^

To change the 5′ UTR length, parts of the *Xenopus* Beta Globin 5′ UTR (5′ – CTTGTTCTTTTTGCAGAAGCTCAGAATAAACGCTCAACTTTGG – 3’) were appended to the endogenous 5′ UTR sequence to achieve the intended length. When shortening the 5′ UTR length, the endogenous sequence is used, truncating the 5′ end to reach the intended length.

For MRPS38/AURKAIP1 the RNA secondary structure sequence was mutated from 5′-ttgagccgcgtcgaggtcgggcttgggaagggtcagcgggaggcCTGagGgcGccGggCgctgcGgcaggCggGccCggGgtCcaGccGagaggCtcAcctggGacCtgtggCcgCcgCccacaGacC-3’ to 5′-ttgagccgcgtcgaggtcgggcttgggaagggtcagcgggaggcCTGagAgcAccAggTgctgcAgcaggTggTccAggTgtTcaAccAagaggTtcAcctggTacTtgtggTcgTcgTccacaAacT-3’.

#### Subcellular localization predictions

DeepLoc2.1,^40^ PSORTII,^72^ SignalP,^74^ TargetP,^73^ and NoD^75^ were used to predict subcellular protein localizations. For all predictions, we used the webserver and default settings to generate the predicted localizations. We note that these prediction programs often struggle to accurately distinguish between nuclear and cytosolic localization. This limitation likely stems from the fact that proteins without specific targeting signals—such as GFP—can freely localize to both compartments. To avoid this ambiguity, we focused our analysis on localizations to non-nuclear organelles.

We selected the strongest translation initiation site for each ORF type (extension, annotated, truncation) per gene prior to any localization predictions. To do this, we selected initiation sites with the highest translation initiation efficiency (ribosome footprint reads at the initiation site from harringtonine ribosome profiling samples normalized to mRNA abundance) from asynchronous HeLa cells.^39^ If an ORF is predicted to be a uORF in one mRNA isoform but an N-terminal extension in another isoform, we selected the transcript that produced the uORF rather than N-terminal extension for the analysis in Figure 1 such that only unambiguous N-terminal extensions were included.

To quantify localization changes and generate the Sankey plot, we compared the predicted localization of the annotated isoform with that of the alternative isoform. If an alternative isoform had multiple predicted localizations, each distinct transition was counted separately. For example, if the annotated isoform was predicted to localize to the mitochondria, whereas the alternative isoform localized to both the cytoplasm and nucleus, we recorded two transitions: (1) mitochondria to cytoplasm and (2) mitochondria to nucleus. Similarly, if the annotated isoform was cytosolic and nuclear, and the alternative isoform was mitochondrial, we classified this as (1) cytosol to mitochondria and (2) nucleus to mitochondria. When both isoforms had multiple localizations, all possible transitions were counted. For instance, if the annotated isoform was mitochondrial and nuclear, while the alternative isoform was cytosolic and nuclear, we recorded: (1) mitochondria to cytosol, (2) mitochondria to nucleus, (3) nucleus to cytosol, and (4) nucleus to nucleus.

For isoforms that shared the same localization we recorded no transitions across organelles. For example, if both isoforms localized cytoplasm and nucleus then we recorded 1) cytosol to cytosol and 2) nucleus to nucleus.

For the analysis of TRNT1, the sequences from Ensembl (human ENST00000251607; rhesus monkey ENSMFAT00000093651; mouse ENSMUST00000057578; frog ENSXETT00000066167; fish ENSDART00000113706; fly FBtr0301574) was used in the predictions. The current WBcel235 annotation has the “truncated” TRNT1 isoform as “annotated” due to a misannotation (hpo-31; F55B12.4.1). For our predictions, we identified the longest open reading frame and designated it as the “annotated” ORF.

When plotting predicted probability of localization for TRNT1 using DeepLoc2.1 (Figures 3J and S4E), we took the average predicted probability for nucleus and cytosol prior to plotting. This is because by default (for example GFP) most proteins are cytosolic/nuclear. We analyzed the data comparing mitochondrial vs. nuclear localization, as well as mitochondrial vs. cytosolic localization, and did not observe any significant differences so decided to plot predicted mitochondrial vs cytosolic/nuclear localization.

#### Live-cell imaging of dox-inducible GFP cell lines

The GFP transgene was induced by addition of 1 μg/mL dox for 24 hours prior to imaging. Cells on glass bottom plates were incubated in 0.1 μg/mL Hoescht for >30 minutes. Images were taken on the Deltavision Ultra (Cytiva) system using a 60x/1.42NA objective. 8 μm images were taken with z-sections of 0.2 μm. All images except for mitochondrial translation assays presented were deconvolved and max projected. When quantitatively comparing intensities between images, the images were scaled equivalently and raw images were used.

#### Immunofluorescence

For the analysis of extended MRPS38 knockouts, polyclonal cell populations were examined 9 days post-infection (6 day post-selection), a time point at which not all cells have undergone complete knockout. Polyclonal populations were used because complete knockout of extended MRPS38 is lethal. Cells were seeded on poly-L-lysine coated coverslips and fixed in PBS + 4% formaldehyde at room temperature for 15 minutes. To pre-extract cytosolic signal the plate of cells were placed on ice and incubated with 10 mM HEPES, 10 mM NaCl, 5 mM KCl, 300 mM sucrose, and 0.015% digitonin for 2 minutes. Cells were then washed with 10 mM HEPES, 10 mM NaCl, 5 mM KCl, 300 mM sucrose then fixed with PBS + 4% formaldehyde at room temperature for 15 minutes. Following fixation, cells were washed 3 times with PBS + 0.1% Triton X-100 and blocked in AbDil for 30 minutes to 1 hour. Following blocking, cells were stained at room temperature for 1 hour with ChromoTek GFP-Booster Alexa Fluor 488 (1:1000, Proteintech, gb2AF488) and α-COXIV (1:1000, Cell Signaling, 119675S) diluted in AbDil. For MRPS38/AURKAIP1 immunofluorescence, cells were incubated with anti-AURKAIP1/MRPS38 (1:1000, Thermo, PA5–56869) overnight at 4°C. Cells were washed with PBS + 0.1% Triton X-100, 3 times then incubated in secondary antibody diluted in AbDil for 1 hour at room temperature. After secondary, cells were stained in 1 μg/mL Hoescht for 15 minutes at room temp, then washed 3 times with PBS + 0.1% Triton X-100 before mounting in PPDM (0.5% p-phenylenediamine and 20 mM Tris-Cl, pH 8.8, in 90% glycerol) and sealed with nail polish.

For the analysis of extended MRPS38 knockouts, polyclonal cell populations were examined six days post-infection, a time point at which not all cells have undergone complete knockout. Polyclonal populations were used because complete knockout of extended MRPS38 is lethal.

#### Start codon conservation analysis

PhyloP scores from alignments of 99 vertebrates to human genome (100way) were used in analyses. For start codons, the average PhyloP score for the 3 nucleotides were used in analyses. MitoCarta3^80^ was used to define genes that are mitochondrial. We note that a change from a CUG to UUG or another non-AUG start codon between organisms may have a low PhyloP score but the usage of the codon may still be used across organisms.

#### Endogenous Tagging of TGGT1_315810 (TRNT1) in *T. gondii*

Type I RH parasites were grown in human foreskin fibroblasts (HFFs, ATCC SCRC-1041) and maintained in DMEM (GIBCO) supplemented with 2 mM glutamine, 10% inactivated fetal bovine serum (IFS), and 10 μg/ml gentamicin. TGGT1_315810 was endogenously tagged in *T. gondii* using a previously described high-throughput (HiT) tagging system.^81^ Briefly, a cutting unit targeting the 3′ end of the coding sequence of TGGT1_315810 was cloned into a plasmid carrying a V5-TEV-mCherry-HA payload. Prior to transfection, 50 μg of this plasmid was linearized with BsaI-HF V2 (NEB) and dialyzed for 1 h. The linearized plasmid was co-transfected with a Cas9 expression vector into RHΔku80Δhxgprt parasites. During transfection, parasites were resuspended in transfection solution containing 0.15 mM CaCl2, 2 mM ATP, 5 mM glutathione, 15 – 60 μg of DNA, and cytomix (10 mM KPO4, 120 mM KCl, 5 mM MgCl2, 25 mM HEPES, 2 mM EDTA, 2 mM ATP, and 5 mM glutathione) up to 400 μl. Parasites were transfected by electroporation with two 150 μs pulses at 100 ms intervals at 1700 V. One day post transfection, parasites were selected with 25 μg/mL mycophenolic acid and 50 μg/mL xanthine pH-balanced with 0.3 N HCl. Correct integration of the construct was validated by PCR.

#### Immunofluorescence of tagged TGGT1_315810

Parasites were infected onto a confluent monolayer of HFFs on coverslips. One day post infection, wells were washed with PBS and fixed with a 4% formaldehyde solution at room temperature for 20 min. Wells were washed 3x with PBS, permeabilized with 0.1% triton for 8 min, and washed again 3x with PBS. Wells were blocked for 10 min with 5% goat serum/5% fetal bovine serum in PBS. Wells were incubated with anti-HA (Biolegend #901501; 1:1000 dilution) and anti-HSP70 (a gift from Dominique Soldati; 1:1000 dilution) primary solution for 30 min at room temperature. Wells were then washed 3x with PBS, blocked, and stained with secondary solution (1:1000 anti-rabbit Alexa Fluor 594 ThermoFisher A11037, and 1:1000 anti-mouse Alexa Fluor 488 ThermoFisher A11029) for 30 min at room temperature. Wells were then washed 3x with PBS, dipped twice in dH2O, and mounted on glass slides with Prolong Diamond Antifade Mountant.

#### Immunoprecipitations

Polyclonal GFP antibodies was coupled to Protein A beads as described in Cheeseman and Desai.^82^

For MRPS38 GFP IP experiments to map N-terminal extension peptides, cells were washed in PBS and resuspended 1:1 in 50 mM HEPES pH7.4, 1 mM EGTA, 1 mM MgCl_2_, 100 mM KCl, 10% glycerol then flash frozen in liquid nitrogen. Cells were thawed after addition of an equal volume of 75 mM HEPES pH 7.4, 1.5 mM EGTA, 1.5 mM MgCl_2_, 150 mM KCl, 15% glycerol, 0.075% NP-40, 1X Complete EDTA-free protease inhibitor cocktail (Roche, 4693159001), and 1 mM PMSF. Cells were lysed by sonication and cleared by centrifugation at 21000× g for 30 minutes at 4°C. The supernatant was mixed with Protein A beads coupled to rabbit anti-GFP antibodies and rotated end-over-end at 4°C for 2 hours. Beads were rinsed twice in wash buffer (50 mM HEPES pH 7.4, 1 mM EGTA, 1 mM MgCl_2_, 100 mM KCl, 10% glycerol, 0.05% NP-40, 1 mM DTT, 10 μg/mL leupeptin/pepstatin/chymostatin). Beads were then washed 3 times in wash buffer by rotating for 5 minutes at 4°C. Bound protein was eluted with 100 mM glycine pH 2.6, precipitated by addition of 1/5th volume TCA at 4°C overnight. TCA precipitant was washed 3 times with −20°C acetone and dried using a speed vac.

For quantitative MRPS38 IP-MS experiments involving extended MRPS38, cells were processed as described above, with no detergent included in the lysis buffer to maintain mitochondrial integrity. This approach allowed for the preservation of intact mitochondria, which were then spun out to limit *in vitro* re-association of nucleolar MRPS38 with mitochondrial proteins.

#### Mass spectrometry sample preparation

Proteins underwent digestion and purification following a modified S-trap protocol (Protifi, V4.7). The TCA precipitate was dissolved in a lysis buffer composed of 5% SDS and 50 mM TEAB at pH 8.5, then heated to 95°C for 10 minutes with 20 mM DTT to denature the proteins. Subsequently, samples were treated with 40 mM iodoacetamide for 30 minutes at room temperature to alkylate cysteine residues. The mixture was then acidified to a final concentration of 2.5% phosphoric acid. An S-trap binding/wash buffer was added in a volume six times that of the sample, and the solution was loaded onto S-trap mini columns. Centrifugation at 4000×g for 30 seconds was performed, followed by four washes with 150 μL of the binding/wash buffer, each with the same centrifugation conditions. After the final wash, columns were dried by spinning at 4000×g for 1 minute. On-column digestion was carried out overnight at 37°C in a humidified incubator using 20 μL of 50 mM TEAB (pH 8.5) containing 1 μg of trypsin. Peptides were eluted sequentially with 40 μL of 50 mM TEAB containing 0.2% formic acid, followed by a solution of 50% acetonitrile with 0.2% formic acid. The eluted peptides were quantified using the Thermo fluorescent peptide quantification kit, rapidly frozen in liquid nitrogen, and then lyophilized.

#### TMT labelling and peptide fractionation

Approximately 1.5 μg of trypsin-digested peptides were dissolved in 50 mM triethylammonium bicarbonate (TEAB) at pH 8.5. Each sample was then labeled with the TMT10plex Isobaric Labeling Reagent Set (Thermo Fisher Scientific, 90111), using a label-to-peptide weight ratio of 30:1, and incubated for 1 hour at room temperature. To quench the labeling reaction, 0.2% hydroxylamine was added, and the mixture was left at room temperature for 15 minutes. The labeled samples were combined on ice, rapidly frozen, and lyophilized. The pooled TMT-labeled peptides were subsequently purified and fractionated using the Pierce High pH Reversed-Phase Peptide Fractionation Kit (Thermo Fisher Scientific, 84868), following the manufacturer’s guidelines for TMT experiments. After fractionation, fractions 1+2, 3+4, 5+6, and 7+8 were pooled, flash-frozen, and lyophilized.

#### Mass spectrometry data acquisition

Lyophilized peptides were reconstituted in 0.2% formic acid to achieve a final concentration of 250 ng/μL. Mass spectrometric analysis was conducted using a Thermo Scientific Orbitrap Exploris 480 mass spectrometer, equipped with a FAIMS Pro interface, and coupled to an EASY-nLC chromatography system. Peptide separation was performed on a 25 cm analytical column (PepMap RSLC C18, 3 μm, 100Å, 75 μm inner diameter). The chromatographic gradient was executed at a flow rate of 300 nL/min as follows: 6–21% solvent B over 41 minutes, 21–36% B for 20 minutes, 36–50% B for 10 minutes, 50–100% B over 15 minutes, followed by column washing and re-equilibration steps. The mass spectrometer and FAIMS interface operated in positive ion mode with a spray voltage of 1800 V and an ion transfer tube temperature set to 270°C. Standard FAIMS resolution settings were applied, utilizing compensation voltages of −50 V and −65 V for the first injection, and −40 V and −60 V for the second. Full scan spectra were acquired in profile mode at a resolution of 120,000, covering a mass-to-charge (m/z) range of 350–1200. The instrument employed an automatic determination of maximum fill time, standard automatic gain control (AGC) target, an intensity threshold of 5×10^3^, selected charge states of +2 to +5, and a dynamic exclusion duration of 30 seconds.

#### Mass spectrometry analysis

Raw data files were processed using Proteome Discoverer version 2.4 (Thermo Fisher Scientific) to identify proteins and peptides. The Sequest HT search engine was employed, using the Homo sapiens protein database (UP000005640) supplemented with EGFP sequences and the MRPS38 N-terminal extension sequence when appropriate. The search parameters allowed for a maximum of two missed trypsin cleavage sites. Mass tolerances were set to 10 ppm for precursor ions and 0.02 Da for fragment ions. The analysis considered several post-translational modifications: dynamic phosphorylation (+79.966 Da on serine, threonine, or tyrosine), dynamic oxidation (+15.995 Da on methionine), dynamic acetylation (+42.011 Da at the N-terminus), dynamic loss of methionine (−131.04 Da at the N-terminal methionine), dynamic loss of methionine with acetylation (−89.03 Da at the N-terminal methionine), and static carbamidomethylation (+57.021 Da on cysteine). For TMT experiments, static modifications included TMT6plex (+229.163 Da at any N-terminus) and TMT6plex (+229.163 Da on lysine residues). Isotope correction factors for TMT 10plex were applied according to the manufacturer’s specifications (Thermo Fisher; product number 90111, lot number VK306786). Peptide identifications were filtered using Percolator to achieve a false discovery rate (FDR) of no more than 0.01. For semi-quantitative IPs, a fixed-value PSM validator was used to filter peptides, retaining only those with a delta Cn ≤ 0.05. Peptides mapping to the MRPS38 N-terminal extension were observed, independent of peptide filtering methods.

#### *In vitro* transcription, capping, polyadenylation, and purification

Plasmids containing the reporter gene with a C-terminal GFP tag were amplified via PCR with an oligo with the T7 promoter (5′-TAA TACGACTCACTATAGGG-3’) in 5′ oligo to generate templates for *in vitro* transcription. The resulting PCR products were digested with DpnI to remove plasmid template, purified using EconoSpin^™^ All-in-1 Mini Spin Columns (Epoch Life Science, 1920–250) and eluted with RNase-free water. For transcription, 1 μg of purified PCR product was used in an *in vitro* reaction with the HiScribe T7 High Yield RNA Synthesis Kit (NEB, E2040S) in the presence of 20 U/ml SUPERaseIn. To remove free nucleotides and abortive transcripts, the reaction was passed through P30 columns (Bio-Rad, 732–6251), followed by phenol-chloroform extraction for further purification. The transcribed RNA was then capped using the Vaccinia Capping System (NEB, M2080S) according to the manufacturer’s instructions, purified again using P30 columns, and subjected to phenol-chloroform extraction. To add a poly(A) tail, *Escherichia coli* Poly(A) Polymerase (NEB, M0276) was used, followed by another round of P30 column purification and phenol-chloroform extraction. The final capped and polyadenylated RNA was quantified using a Nanodrop, aliquoted, flash-frozen, and stored at −80 °C.

#### mRNA transfections for luciferase assays

Cells were cultured in 24-well plates until reaching approximately 80% confluency before transfection. Transfections were performed using Lipofectamine MessengerMAX (Thermo Fisher, LMRNA008) following the manufacturer’s instructions, with 500 ng of mRNA per well and 1 μL of Lipofectamine MessengerMAX. For luciferase assays, cells were harvested 3 hours after transfection.

#### Luciferase assays

Luciferase assays were conducted with the Dual-Luciferase Reporter Assay System (Promega, E1980) following the manufacturer’s instructions. Luminescence was measured using the GloMax Discover Microplate Reader (Promega).

#### mRNA transfections for live-cell imaging

Cells were grown to ~80% confluency in a 12-well plate. Cells were transfected with Lipofectamine MessengerMAX with 1000 ng of mRNA and 2 μl of Lipofectamine MessengerMAX per well. Cells were dislodged from the plate using PBS + 5 mM EDTA and reseeded at 20% density in a 12 well glass bottom plate. 16 hours after reseeding, cells were incubated with 0.1 μg/mL Hoescht for 30 minutes then live imaged as described above.

#### 5′ Rapid Amplification of cDNA Ends (RACE)

RACE reactions were performed with Template Switching RT Enzyme Mix (NEB, M0466) according to manufacturer instructions. 1ug total RNA was used for cDNA preparation for all reactions. Reverse-transcription primers were annealed with RNA for 5 minutes at 70°C, followed by reverse transcription and template switching using a template-switching oligo (5′-GCTAATCATTGCAAGCAGTGGTATCAACGCAGAGTACATrGrGrG-3’). For reverse transcription, samples were incubated for 90 minutes at 42°C and 5 minutes at 85°C. PCR amplification was performed with Q5 Hot Start High-Fidelity Master Mix (2X) (NEB, M0494) using 5–20% of cDNA product from reverse-transcription depending on target transcript abundance. To increase specificity, nested primers internal to the reverse-transcription primers were used for PCR amplification, along with a touchdown-PCR protocol with initial cycles of high annealing temperatures (72°C, 70°C) to enrich for target-specific products. PCR-amplified products were run on 1.8% agarose gels for imaging or purified with Zymo DNA Clean and Concentrator kits (D4004) for sequencing analysis using the MGH CCIB DNA core complete amplicon sequencing service. The sequencing reads were assembled de novo, and the number of reads with a unique 5′ end was quantified in the 5′ RACE plots.

#### Lentivirus generation

To generate lentivirus, HEK293T cells at ~80% confluency in a 6 well plate was transfected with 1.2 μg lentiCas9-sgRNA transfer plasmid with puromycin resistance or mCherry, 1 μg psPAX2 packaging plasmid, and 0.4 μg vsFULL envelope plasmid using Xtremegene-9 (Roche). After 16 hours post transfection, the media was swapped. After an additional 24 hours, the lentivirus in the supernatant was collected and stored at −80°C.

#### Competitive growth assays

Fluorescent (eBFP2 or mCherry) HeLa cells were infected with lentivirus carrying Cas9-sgRNA targeting a specific MRPS38 locus (5′-GGCGGCGGCCACAGGTCCCA-3’, 5′-GGGCCCGGGGTCCAGCCGAG-3’, and 5′-AGCGGGAGGCCTGAGGGCGC-3’ for isoform specific guides and 5′-AGGAACGGCCCTCAACAGCT-3’, 5′-GTGTCCGCCCAGCCAGATAG-3’, 5′-GGAAGCTGGTGAAGAAGACG-3’ for all MRPS38 isoforms). Cells at ~90% confluency were spinfected with lentivirus in the presence of 10 μg/mL polybrene. After 16 hours, the media was swapped. After an additional 24 hours, cells were moved into a new plate and 0.4 μg/mL puromycin was added for 72 hours. After the selection was complete, equal amounts of control eBFP cells were mixed with mCherry cells with Cas9 targeting a specific locus. The population of eBFP:mCherry cells were monitored every 3 days using the BD FACSymphony A1 Cell Analyzer (BD Biosciences).

HeLa cells were infected with lentivirus carrying Cas9-sgRNA targeting specific regions of TRNT1 (Mitochondrial only isoforms: gRNA1: 5′-ACAGGCCAGTGCTGAACCGT-3’ and gRNA2: 5′-GGTGCCTGTATCATTGGCAC-3’; Both isoforms: gRNA1: 5′-GAAGGACTGAAGAGTCTGAC-3’ and gRNA2: 5′-AACTTGCTCTCACCTATATA-3’) or the HS1 locus (5′-GCCGATGGTGAAGTGGTAAG-3’), and mCherry or BFP. Cells at ~90% confluency in a 6 well plate were spinfected with lentivirus in the presence of 10 μg/mL polybrene. After 16 hours, cells were moved into a 10 cm plate. Cells were grown for another day at 37C, then sorted for mCherry or BFP. After sorting fluorescent cells, the cells were grown for an additional 2 days. Equal amounts of HS1 sgRNA BFP cells were mixed with TRNT1 sgRNA mCherry cells. The population of BFP:mCherry cells were monitored every 3 days using the BD FACSymphony A1 Cell Analyzer (BD Biosciences).

#### Total HPG incorporation assay

Polyclonal isoform specific TRNT1 knockout cells were generated as described above. Translation rates were monitored 6 days after lentiviral transduction (or 4 days after sorting). Cells were grown in 12 well plates, incubated with 500 μM Click-IT L-Homopropargylglycine (HPG) for 30 minutes in standard DMEM. Following this incubation, cells were washed once with DMEM, trypsinized, and fixed with 4% formaldehyde for 15 minutes at room temperature. After fixation, cells were washed with PBSTx (PBS + 0.1% Triton X-100) 3 times and blocked with AbDil overnight. Click chemistry was performed by incubating the cells in 0.1M Tris pH 8, 1 mM CuSO4, 0.1M ascorbic acid, and 5 μM Alex Fluor 647 Azide (Thermo, A10277) for 30 minutes in the dark. After click chemistry, cells were washed 3 times with PBSTx, strained into flow cytometry tubes, and analyzed using BD FACSymphony A1 Cell Analyzer (BD Biosciences). HPG signal in these assay measures the summed translation rates of cytosolic and mitochondrial ribosomes.

#### Mitochondrial translation assay

Polyclonal isoform specific TRNT1 knockout cells were seeded on poly-lysine treated coverslips. Mitochondrial translation rates were monitored 6 days after lentiviral transduction (or 4 days after sorting). Cells were in incubated with 500 μM Click-IT L-Homopropargylglycine (HPG) for 30 minutes in standard DMEM. Following this incubation, cells were washed once with DMEM, once with PBS, then fixed with 4% formaldehyde for 15 minutes at room temperature. After fixation, cells were washed with PBSTx (PBS + 0.1% Triton X-100) 3 times and blocked with AbDil overnight. Click chemistry was performed on the coverslip by incubating the cells in 0.1M Tris pH 8, 1 mM CuSO4, 0.1M ascorbic acid, and 5 μM Alex Fluor 647 azide (Thermo, A10277) for 30 minutes in the dark. After click chemistry, cells were incubated with 0.1 μg/mL Hoescht for 15 minutes, washed 3 times with PBSTx, then mount in PPDM (0.5% p-phenylenediamine and 20 mM Tris-Cl, pH 8.8, in 90% glycerol) and sealed with nail polish. Images were acquired with Deltavision Ultra (Cytiva) system using a 60x/1.42NA objective. Nuclear HPG signal represents product from cytosolic translation whereas mitochondrial signal indicates mitochondrial translation.

#### ClinVar analysis and SwissIsoform

To facilitate the systematic analysis of alternative protein isoforms and their clinical significance, we developed SwissIsoform, a comprehensive computational framework that integrates genomic annotations, ribosome profiling data, and clinical mutation databases to identify and prioritize functionally relevant protein variants. SwissIsoform processes alternative translation start sites from ribosome profiling experiments alongside GENCODE annotations and clinical mutation data from ClinVar, enabling detailed characterization of truncated protein isoforms and their relationship with disease-associated variants.

The SwissIsoform pipeline selects the highest efficiency translation initiation site for a gene if there are multiple alternative start sites are used (Figure S1D). The pipeline systematically identifies annotated-truncation pairs by mapping alternative translation initiation sites to annotated transcript features. For each pair, the framework performs comprehensive mutation analysis, querying clinical databases to identify variants within regions between the annotated and truncation start site and categorizing them by molecular impact (missense, nonsense, frameshift). The pipeline quantifies mutation burden across different variant types and generates detailed visualizations showing transcript architecture, alternative start sites, and mutation distributions, revealing potential pathogenic mechanisms associated with alternative translation events.

To enable functional prioritization of extensive alternative isoform datasets, SwissIsoform generates standardized protein sequences for both annotated and truncated variants, with optional integration of clinical mutations. The generation of protein sequences will use the most highly expressed mRNA isoform in HeLa cells for each translation initiation site. These sequences are formatted for direct compatibility with protein language model-based prediction tools, including DeepLoc for subcellular localization analysis. The framework supports both fast (ESM1b) and accurate (ProtT5) prediction modes, enabling researchers to balance computational efficiency with prediction precision based on their specific requirements.

SwissIsoform’s analysis pipeline culminates in comprehensive summary reports that identify genes where alternative isoforms exhibit altered subcellular localization compared to canonical proteins, highlighting variants where truncation or mutation events may disrupt cellular trafficking and function. This integrated approach enables researchers to systematically prioritize clinically relevant alternative isoforms by combining mutation burden analysis with predicted functional consequences, streamlining the identification of high-priority candidates for experimental validation and therapeutic targeting.

To analyze “missense” mutations that affect alternative start codons, we used the ClinVar variant table released on March 31^st^, 2024. We used BEDTools intersect^76^ to identify alleles that overlap with alternative start codons.

### QUANTIFICATION AND STATISTICAL ANALYSIS

For all experiments, n indicates biological replicates and at least 2 biological replicates were performed for each experiment. For bar graphs, the mean of biological replicates is plotted and error bars indicate standard error of the mean. Peptide identifications for mass spectrometry experiments were filtered using Percolator to achieve an FDR of no more than 0.01. ANOVA was used for statistical test for quantitative proteomics using Proteome Discoverer. Details for number of replicates and statistical test can be found in the figure legends and STAR Methods.

## Supplementary Material

MMMC1

MMMC3

MMMC2

MMMC4

MMMC6

MMC5

SUPPLEMENTAL INFORMATION

Supplemental information can be found online at https://doi.org/10.1016/j.molcel.2025.10.013.

## Figures and Tables

**KEY RESOURCES TABLE:** 

REAGENT or RESOURCE	SOURCE	IDENTIFIER

Antibodies		

ChromoTek GFP-Booster AlexaFluor 488	Proteintech	gb2AF488; RRID: AB2827573
Rabbit polyclonal GFP	Cheeseman lab	N/A
Rabbit polyclonal MRPS38/AURKAIP1	Thermo Fisher	PA5-56869; RRID: AB_2638401
Mouse mAb COXIV	Cell Signaling	11967S; RRID: AB_2797784
Mouse mAb HA	BioLegend	901501; RRID: AB_2565006
Rabbit polyclonal HSP70 (*T. Gondii*)	Pino et al.^71^	N/A

Chemicals, peptides, and recombinant proteins		

L-Homopropargylglycine (HPG)	Thermo Fisher	C10186
5-Ethynyluridine (5-EU)	Vector Laboratories	CCT-1261-100
TRI Reagent solution	Thermo Fisher	AM9738
Phenol chloroform	Sigma	P2069
Chloroform	Sigma	CX1055-6
Trichloroacetic acid	Sigma	T6399
Iodoacetamide	Sigma	I1149
Lipofectamine 2000	Invitrogen	11668027
Lipofectamine MessengerMax	Invitrogen	LMRNA008
Q5 High-Fidelity 2x Master Mix	NEB	M0492L
Gibson Assembly Master Mix	NEB	E2611L
DNA Clean and Concentrator-5	Zymo Research	D4004

Critical commercial assays		

Maxima First Strand cDNA Synthesis Kit for RT-qPCR	Thermo Fisher	K1671
S-trap micro columns	ProtiFi	C02-micro-80
TMT10plex^™^ Isobaric Label Reagent Set	Thermo Fisher	90110
Pierce High pH Reversed-Phase Peptide Fractionation Kit	Thermo Fisher	84868
HiScribe T7 High Yield RNA Synthesis Kit	NEB	E2040S
Vaccinia Capping System	NEB	M2080S
E. Coli Poly(A) Polymerase	NEB	M0276L
Micro Bio-Spin P-30 columns	Bio-Rad	7326251
Dual-luciferase Reporter Assay System	Promega	E1980
Template Switching RT Enzyme Mix	NEB	M0466

Deposited data		

Mass spec data	This study	PRIDE: PXD062112
Raw images and gel	This study	Mendeley data: https://doi.org/10.17632/m6rj76j5hm.1 https://doi.org/10.17632/nv9jb8f5mg.1 https://doi.org/10.17632/r3w7hr7j36.1

Experimental models: Cell lines		

HeLa cells	Cheeseman lab	N/A
HEK293T cells (for lentivirus generation)	Cheeseman lab	N/A
HFF cells (for *T. gondii* culturing)	ATCC	SCRC-1041

Experimental models: Organisms/strains		

*Toxoplasma Gondii* RHΔku80Δhxg	ATCC	PRA-319

Oligonucleotides		

RACE template switching oligo; 5′-GCTAATCATTGCAAGCAGTGGTATCAACGCAGAGTACATrGrGrG-3′	This study	oJL1137
RACE PCR oligo; 5′-CATTGCAAGCAGTGGTATCAAC-3′	This study	OJL1152
Human TRNT1 RACE reverse transcription oligo; 5′-CCGCATCTTTCTGCCAGTCAG-3′	This study	oYT217
Human TRNT1 RACE nested PCR primer; 5′-CCATCAGTGGTGACATCAATCCG-3′	This study	oYT218
Mouse TRNT1 RACE reverse transcription oligo; 5′-GAAGCCTGGCAGTGATAGTCC-3′	This study	oYT219
Mouse TRNT1 RACE nested PCR primer; 5′-CCTTTGTTGTTGATCATGCGAATGCC-3′	This study	oYT220
Yeast TRNT1 RACE reverse transcription oligo; 5′-GCTCCATATTTGGCGTAATGTTGTTGC-3′	This study	oYT223
Yeast TRNT1 RACE nested PCR primer; 5′-CTCACCTGACATCACATTGATGGC-3′	This study	oYT224
AURKAIP1/MRPS38 PCR primers for IVT template; 5′-TAATACGACTCACTATAGGG-3′; 5′-CATGTCTGGATCTACGCCCGGATCCCTCGAGGTCG-3′	This study	oJL555 and KXU030
WT PPA2 PCR primers for IVT template; 5′-TAATACGACTCACTATAGGGGTCATTGACGCCATGAGCGCGCTGCTGCGGCTGCTGCG-3’; 5′-TCCCTCGAGGTCGACGAATTCTTACTTGTACAGCTCGTCCATGC-3′	This study	oJL1506 and oJL1122
PPA2 L4fs PCR primers for IVT template; 5′-TAATACGACTCACTATAGGGGTCATTGACGCCATGAGCGCGCTGTGCGGCTGCTGCG-3′; 5′-TCCCTCGAGGTCGACGAATTCTTACTTGTACAGCTCGTCCATGC-3′	This study	oJL1489 and oJL1122

Recombinant DNA		

TRE3G: Annotated AKR7A2-EGFP	This study	pJL597 (cJL409)
TRE3G: Truncated AKR7A2-EGFP	This study	pJL598 (cJL410)
TRE3G: Annotated ALDH9A1-EGFP	This study	pJL806 (cJL565)
TRE3G: Truncated ALDH9A1-EGFP	This study	pJL807 (cJL566)
TRE3G: Annotated C15orf40-EGFP	This study	pJL804 (cJL559)
TRE3G: Truncated C15orf40-EGFP	This study	pJL805 (cJL560)
TRE3G: Annotated CHCHD1-EGFP	This study	pJL831 (cJL574)
TRE3G: Truncated CHCHD1-EGFP	This study	pJL832 (cJL575)
TRE3G: Annotated CMPK1-EGFP	This study	pJL863 (cJL604)
TRE3G: Truncated CMPK1-EGFP	This study	pJL864 (cJL605)
TRE3G: Annotated DPH5-EGFP	This study	pJL587 (cJL399)
TRE3G: Extended DPH5-EGFP	This study	pJL588 (cJL400)
TRE3G: Annotated ERGIC3-EGFP	This study	pJL884 (cJL622)
TRE3G: Annotated FAAH2-EGFP	This study	pJL906 (cJL637)
TRE3G: Truncated FAAH2-EGFP	This study	pJL907 (cJL638)
TRE3G: Annotated FH-EGFP	This study	pJL592 (cJL404)
TRE3G: Truncated FH-EGFP	This study	pJL593 (cJL405)
TRE3G: Annotated GADD45GIP1-EGFP	This study	pJL622 (cJL428)
TRE3G: Truncated GADD45GIP1-EGFP	This study	pJL623 (cJL429)
TRE3G: Annotated GARS-EGFP; optimal M1 Kozak	This study	pJL845 (cJL584)
TRE3G: Truncated GARS-EGFP; M1I (AUG>AUU)	This study	pJL708 (cJL493)
TRE3G: Annotated GLRX2-EGFP	This study	pJL908 (cJL639)
TRE3G: Truncated GLRX2-EGFP	This study	pJL909 (cJL640)
TRE3G: 5UTR + CDS GSR-EGFP	This study	pJL841 (cJL581)
TRE3G: Truncated GSR-EGFP	This study	pJL843 (cJL582)
TRE3G: Annotated MRPS38-EGFP	This study	pJL144 (cJL95)
TRE3G: Extended MRPS38-EGFP	This study	pJL202 (cJL136)
TRE3G: Annotated MTPN-EGFP	This study	pJL922 (cJL651)
TRE3G: Extended MTPN-EGFP	This study	pJL923 (cJL652)
TRE3G: Annotated NAXE-EGFP	This study	pJL839 (cJL579)
TRE3G: Truncated NAXE-EGFP	This study	pJL840 (cJL580)
TRE3G: NTHL1 5UTR-CDS-EGFP	This study	pJL827 (cJL576)
TRE3G: Truncated NTHL1-EGFP	This study	pJL829 (cJL572)
TRE3G: Annotated PCBD2-EGFP	This study	pJL820 (cJL567)
TRE3G: Truncated PCBD2-EGFP	This study	pJL821 (cJL568)
TRE3G: Annotated PNPO-EGFP	This study	pJL768 (cJL532)
TRE3G: Truncated PNPO-EGFP	This study	pJL578 (cJL390)
TRE3G: Annotated PPA2-EGFP	This study	pJL904 (cJL635)
TRE3G: Truncated PPA2-EGFP	This study	pJL905 (cJL636)
TRE3G: Annotated PTMS-EGFP	This study	pJL572 (cJL384)
TRE3G: Extended PTMS-EGFP	This study	pJL573 (cJL385)
TRE3G: Annotated RFK-EGFP	This study	pJL590 (cJL402)
TRE3G: Extended RFK-EGFP	This study	pJL591 (cJL403)
TRE3G: Annotated SPAST-EGFP	This study	pJL700 (cJL472)
TRE3G: Extended SPAST-EGFP	This study	pJL699 (cJL471)
TRE3G: Annotated TOP3A-EGFP	This study	pJL679 (cJL456)
TRE3G: Extended TOP3A-EGFP	This study	pJL678 (cJL455)
TRE3G: TRNT1 5UTR-CDS-EGFP, optimal M1 Kozak	This study	pJL641 (cJL439)
TRE3G: Truncated TRNT1-EGFP	This study	pJL580 (cJL392)
TRE3G: Annotated UXS1-EGFP	This study	pJL868 (cJL619)
TRE3G: Truncated UXS1-EGFP	This study	pJL869 (cJL629)
TRE3G: Annotated ACBD6-EGFP	This study	pJL789 (cJL551)
TRE3G: Extended ACBD6-EGFP	This study	pJL788 (cJL550)
TRE3G: Annotated BAG3-EGFP	This study	pJL761 (cJL531)
TRE3G: Extended BAG3-EGFP	This study	pJL760 (cJL530)
TRE3G: Annotated C12orf10-EGFP	This study	pJL815
TRE3G: Truncated C12orf10-EGFP	This study	pJL796 (cJL554)
TRE3G: Annotated C1QBP-EGFP	This study	pJL871 (cJL599)
TRE3G: Extended C1QBP-EGFP	This study	pJL870 (cJL606)
TRE3G: Annotated CBR1-EGFP	This study	pJL801 (cJL558)
TRE3G: Extended CBR1-EGFP	This study	pJL800 (cJL557)
TRE3G: Annotated EGLN3-EGFP	This study	pJL625 (cJL431)
TRE3G: Extended EGLN3-EGFP	This study	pJL626 (cJL432)
TRE3G: Annotated LAGE3-EGFP	This study	pJL786 (cJL548)
TRE3G: Truncated LAGE3-EGFP	This study	pJL787 (cJL549)
TRE3G: Annotated LSM2-EGFP	This study	pJL567 (cJL379)
TRE3G: Extended LSM2-EGFP	This study	pJL568 (cJL380)
TRE3G: Annotated PCSK9-EGFP	This study	pJL595 (cJL407)
TRE3G: Extended PCSK9-EGFP	This study	pJL596 (cJL408)
TRE3G: Annotated PDGFA-EGFP	This study	pJL614 (cJL423)
TRE3G: Extended PDGFA-EGFP	This study	pJL615 (cJL424)
TRE3G: Annotated REXO2-EGFP	This study	pJL584 (cJL396)
TRE3G: Truncated REXO2-EGFP	This study	pJL585 (cJL397)
TRE3G: Annotated RRAGA-EGFP	This study	pJL620 (cJL426)
TRE3G: Extended RRAGA-EGFP	This study	pJL621 (cJL427)
TRE3G: Annotated SORD-EGFP	This study	pJL575 (cJL387)
TRE3G: Extended SORD-EGFP	This study	pJL576 (cJL388)
TRE3G: Annotated STAT3-EGFP	This study	pJL628 (cJL434)
TRE3G: Extended STAT3-EGFP	This study	pJL629 (cJL435)
TRE3G: GARS 5UTR-CDSGARS-EGFP	This study	pJL706 (cJL491)
TRE3G: PNPO 5UTR-CDS-EGFP	This study	pJL577 (cJL389)
TRE3G: MRPS38 5UTR-CDS -EGFP	This study	pJL137 (cJL89)
TRE3G: TRNT1 5UTR CDS-EGFP	This study	pJL579 (cJL391)
TRE3G: AKR7A2^1–29^ N-terminal MTS -EGFP	This study	pJL670 (cJL448)
TRE3G: PTMS extension-EGFP	This study	pJL797 (cJL564)
TRE3G: DPH5 extension-EGFP	This study	pJL671 (cJL528)
TRE3G: DPH5 extension + DPH5^1–25^ (annotated N-term) -EGFP	This study	pJL672 (cJL449)
TRE3G: MRPS38 extension alone-EGFP	This study	pJL147 (cJL98)
TRE3G: FLAG-S-MRPS38-EGFP	This study	pJL206 (cJL298)
TRE3G: MRPS38 21aa extension-EGFP	This study	pJL968 (cJL675)
TRE3G: MRPS38 14aa extension-EGFP	This study	pJL969 (cJL676)
TRE3G: MRPS38 7aa extension-EGFP	This study	pJL970 (cJL677)
TRE3G: NLS TRNT1 masking reporter	This study	pJL991 (cJL497)
TRE3G: FAAH2^1–55^-EGFP	This study	pJL920 (cJL649)
TRE3G: FAAH2^56–96^-EGFP	This study	pJL954 (cJL670)
TRE3G: FLAG-S-truncated FAAH2^56–519^-EGFP	This study	pJL921 (cJL650)
TRE3G: FAAH2^24–519^	This study	pJL972 (cJL679)
TRE3G: TRNT1^1–29^ N-term MTS-EGFP	This study	pJL667 (cJL446)
TRE3G: PNPO^1–31^ N-term MTS-EGFP	This study	pJL893 (cJL631)
TRE3G: GSR^1–43^ N-term MTS-EGFP	This study	pJL894 (cJL632)
TRE3G: CMPK1^1–31^ N-term MTS-EGFP	This study	pJL895 (cJL643)
TRE3G: NAXE^1–51^ N-term MTS-EGFP	This study	pJL896 (cJL633)
TRE3G: RFK extension alone-EGFP	This study	pJL654 (cJL443)
TRE3G: MRPS38^1–50^ annotated N-term MTS-EGFP	This study	pJL148 (cJL99)
TRE3G: MRPS38 synthetic truncation-EGFP	This study	pJL165 (cJL111)
TRE3G: TOP3A^1–25^ annotated N-term MTS-EGFP	This study	pJL681 (cJL458)
TRE3G: TOP3A extension alone-EGFP	This study	pJL680 (cJL457)
TRE3G: FLAG-S-annotated TOP3A-EGFP	This study	pJL751 (cJL515)
TRE3G: TRNT1 5UTR-CDS-EGFP; M1-stop	This study	pJL658 (cJL445)
TRE3G: Mouse TRNT1 5UTR-CDS-EGFP	This study	pJL875 (cJL601)
TRE3G: Drosophila TRNT1 5UTR-CDS-EGFP	This study	pJL886 (cJL624)
TRE3G: Toxoplasma TRNT1 recombination template	This study	pCG240 (TgCG124)
TRE3G: TRNT1 5UTR-CDS-EGFP; M1 AUG>GUG	This study	pJL657 (cJL444)
TRE3G: Dicty G0271378^1–30^-EGFP (paralog 1)	This study	pJL936 (cJL659)
TRE3G: Dicty G0293504^1–30^-EGFP (paralog 2)	This study	pJL937 (cJL660)
TRE3G: MRPS38 5UTR-CDS-EGFP; M1 AUG>AUA	This study	pJL134 (cJL88)
Control HS1 gRNA	This study	pJL199
MRPS38 extension specific gRNA1	This study	pJL257
MRPS38 extension specific gRNA 2	This study	pJL258
MRPS38 extension specific gRNA3	This study	pJL259
MRPS38 both isoform gRNA1	This study	pJL254
MRPS38 both isoform gRNA 2	This study	pJL255
MRPS38 both isoform gRNA3	This study	pJL256
TRNT1 annotated specific gRNA1	This study	pJL663
TRNT1 annotated specific gRNA2	This study	pJL664
TRNT1 both isoform gRNA1	This study	pJL665
TRNT1 both isoform gRNA2	This study	pJL666
MRPS38 5′ UTR CDS-NLuc-IRES-FLuc; wild type	This study	pJL444
MRPS38 5′ UTR CDS-NLuc-IRES-FLuc; ΔCUG	This study	pKK59
MRPS38 5′ UTR CDS-NLuc-IRES-FLuc; ΔAUG	This study	pJL445
MRPS38 5′ UTR CDS-NLuc-IRES-FLuc; ΔAUG ΔCUG	This study	pKK60
MRPS38 5′ UTR CDS-NLuc-IRES-FLuc; RNA secondary structure mutant	This study	pJL673
TRE3G: MRPS38 5′ UTR CDS-EGFP; RNA secondary structure mutant	This study	pJL164 (cJL135)
TRE3G: ELAC2 5UTR-CDS-EGFP	This study	pJL704 (cJL489)
TRE3G: ELAC2 5UTR-CDS-EGFP; A11fs	This study	pJL978 (cJL688)
TRE3G: ALDH7A1 5UTR-CDS-EGFP	This study	pJL929 (cJL662)
TRE3G: ALDH7A1 5UTR-CDS-EGFP; A26fs	This study	pJL955 (cJL671)
TRE3G: NTHL1 5UTR-CDS-EGFP; L4*	This study	pJL867 (cJL598)
TRE3G: NAXE 5UTR-CDS-EGFP	This study	pJL838 (cJL577)
TRE3G: NAXE 5UTR-CDS-EGFP; S43*	This study	pJL844 (cJL583)
TRE3G: GARS1 5UTR-CDS-EGFP; C41*	This study	pJL849 (cJL586)
TRE3G: PNPO 5UTR-CDS-EGFP; H24fs	This study	pJL850 (cJL588)
TRE3G: PNPO 5UTR-CDS-EGFP; R6W	This study	pJL980 (cJL690)
TRE3G: TRNT1 5UTR-CDS-EGFP; c.74_74ins20/p.Gln25fs Patient 1	This study	pJL852 (cJL590)
TRE3G: TRNT1 5UTR-CDS-EGFP; M1V AUG>GUG	This study	pJL657 (cJL444)
TRE3G: TRNT1 5UTR-CDS-EGFP Leu2fs	This study	pJL917 (cJL648)
TRE3G: TRNT1 5UTR-CDS-EGFP; Leu13fs	This study	pJL916 (cJL647)
TRE3G: TRNT1 5UTR-CDS-EGFP; c.88A>T/p.M30L Patient 2	This study	pJL851 (cJL589)
TRE3G: TRNT1 5UTR-CDS-EGFP; M30V AUG>GUG	This study	pJL642 (cJL440)

Software and algorithms		

SwissIsoform v1.0.0, https://doi.org/10.5281/zenodo.17241986	This study	N/A
DeepLoc2.1	Ødum et al.^40^	N/A
PSORTII	Nakai and Horton^72^	N/A
TargetP	Almagro Armenteros et al.^73^	N/A
SignalP	Almagro Armenteros et al.^74^	N/A
NucleOlar localization sequence Detector	Scott et al.^75^	N/A
PhyloP	Pollard et al.^41^	N/A
ClinVar	Landrum et al.^60^	N/A
BEDTools	Quinlan and Hall^76^	N/A
ProteomeDiscoverer	ThermoFisher	N/A
RNA fold	Lorenz et al.^53^	N/A
Fiji (ImageJ)	Schindelin et al.^77^	N/A
SoftWorx	GE Healthcare	N/A
FACSdiva	BD Biosciences	N/A
FlowJo	BD Biosciences	N/A
AlphaFold3	Cheng et al.^66^	N/A

## Highlights

- Alternate N-terminal isoforms diversify the mitochondrial proteome and its function
- N-terminal masking regulates the localization of alternative N-terminal isoforms
- TRNT1 alternative start codons coincide with mitochondrial endosymbiosis
- Alternative N-terminal “isoform-selective alleles” shape rare human diseases
