# Supplementary material for: Alternative start codon selection shapes mitochondrial function and rare human diseases
Source: Mol Cell. Author manuscript; Available in PMC 2025 Dec 20. (PMC12718117; doi:10.1016/j.molcel.2025.10.013)
Supplement: MMMC1 [file NIHMS2117943-supplement-MMMC1.pdf]

Molecular Cell, Volume *85*

Supplemental information

Alternative start codon selection shapes

mitochondrial function and rare human diseases

Jimmy Ly, Matteo Di Bernardo, Yi Fei Tao, Ekaterina Khalizeva, Christopher J. Giuliano, Sebastian Lourido, Mark D. Fleming, and Iain M. Cheeseman

Molecular Cell, Volume *85*

Supplemental information

Alternative start codon selection shapes mitochondrial function and rare human diseases

Jimmy Ly, Matteo Di Bernardo, Yi Fei Tao, Ekaterina Khalizeva, Christopher J. Giuliano, Sebastian Lourido, Mark D. Fleming, and Iain M. Cheeseman

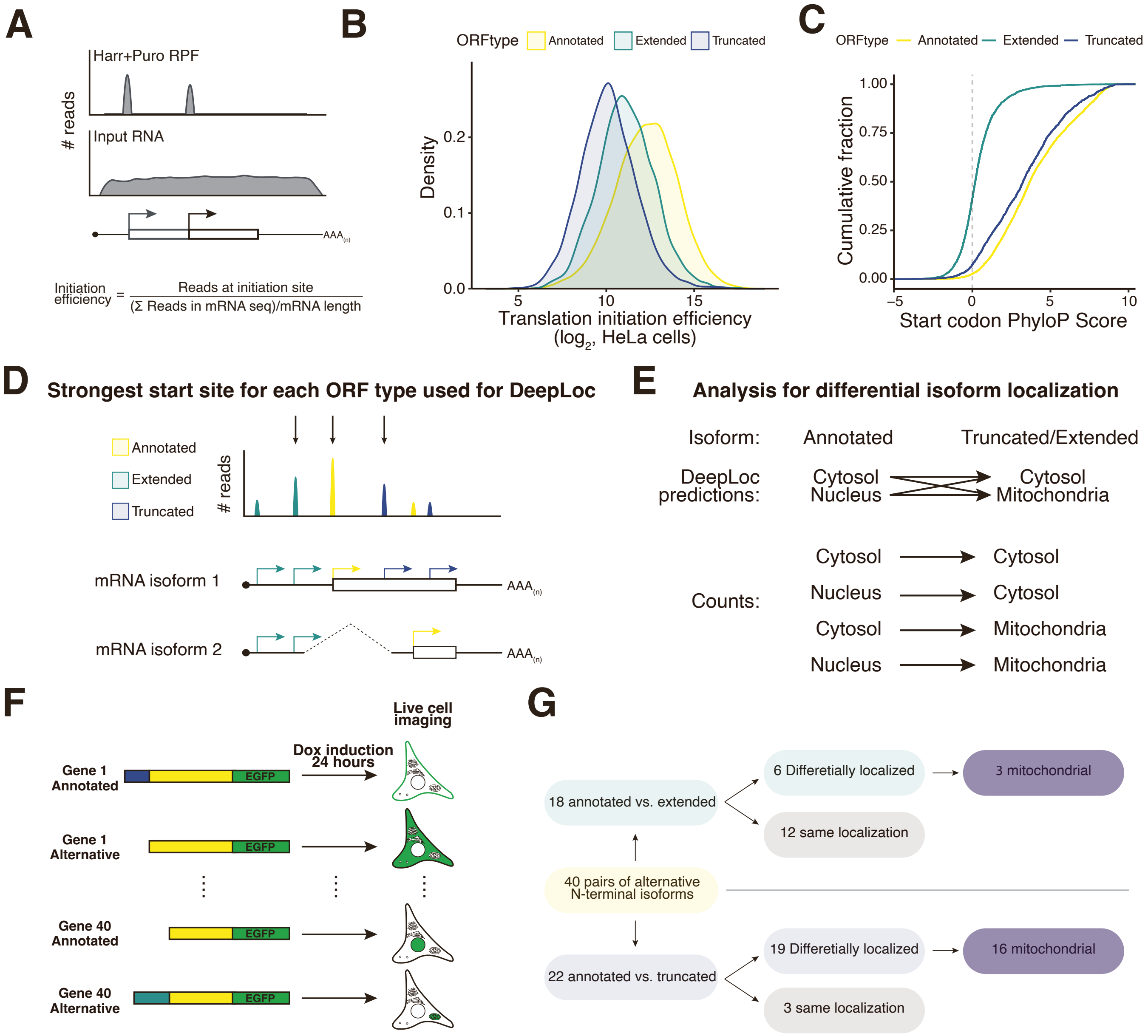

Supplementary figure 1. Predicting and analyzing differentially localized alternative N-terminal isoforms, related to Figure 1. (**A**) Schematic representation of translation initiation site efficiency measurement. (**B**) Histogram showing relative translation initiation efficiency between ORF types from asynchronous HeLa cells [39]. (**C**) CDF plot showing the average PhyloP score of start codons for each ORF types. (**D**) Schematic showing the selection of N-terminal isoform for each ORF type if a gene has multiple translation initiation sites. The start site with the strongest translation initiation efficiency is selected and used for DeepLoc analysis in Figure 1. (**E**) Schematic showing the analysis of DeepLoc output. If the annotated isoform is predicted mitochondrial and the truncated isoform is predicted cytosolic and nuclear, the predicted change for the associated gene is mitochondria to cytosol and mitochondria to nucleus. Additional details described in the Methods. (**F**) Schematic representation of localization screen (40 alternative N-terminal isoforms) to assess the localization of selected alternative N-terminal isoforms. (**G**) Summary of the results from isoform-specific localization screen.

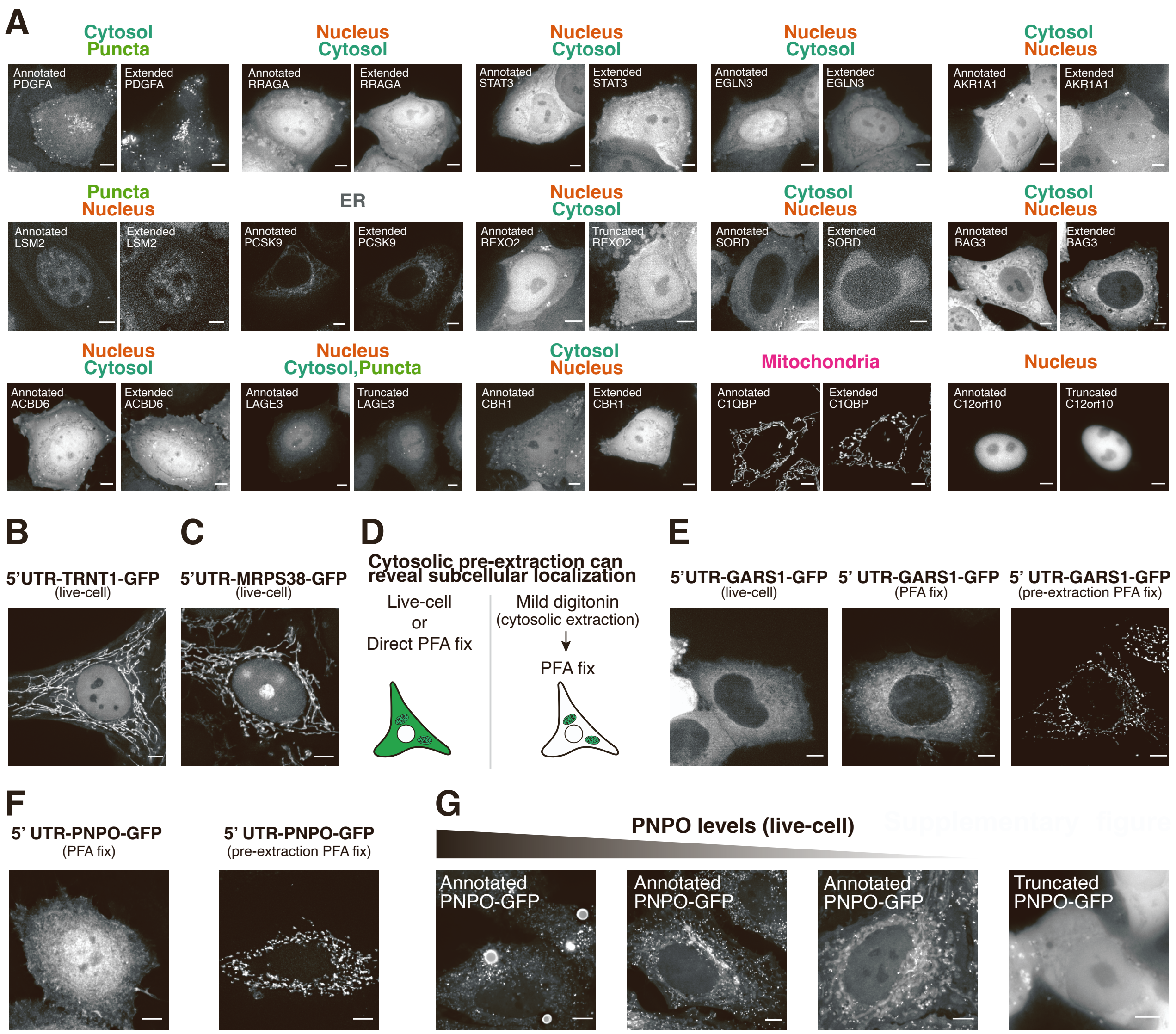

Supplementary figure 2. Additional results and consideration of isoform-specific localization screen, related to Figure 1. (**A**) Live-cell imaging of selected alternative translational isoforms where the same localization was observed. (**B**) Live-cell imaging of TRNT1 5′ UTR – CDS – GFP shows dual localization to nucleus/cytosol and mitochondria. (**C**) Live-cell imaging of MRPS38/AURKAIP1 5′ UTR – CDS – GFP shows dual localization to nucleolus and mitochondria. (**D**) Schematic showing the possible differences between live-cell or direct formaldehyde fixed and cytosolic pre-extracted imaging. Pre-extraction of the cytosolic signal may uncover subcellular localization that is being obscured by the cytosolic signal. (**E**) Localization of GARS1 5′ UTR – CDS – GFP by live-imaging, direct formaldehyde fix, and pre-extraction followed by formaldehyde fixed immunofluorescence. Since the cytosolic isoform of GARS1 is more highly expressed than the mitochondrial isoform, the mitochondrial localization of GARS1 can by clearly observed by pre-extracting cytosolic signal. The localization screen was performed using live-cell imaging. As a result, isoforms with low expression levels or those localized to small organelles may have been overlooked due to dominant cytosolic signal, potentially masking subtler intracellular localization patterns. (**F**) Localization of PNPO 5′ UTR – CDS – GFP by immunofluorescence of direct formaldehyde fix or pre-extraction followed by PFA fixation, highlighting the production of a cytosolic/nuclear and mitochondrial isoform from the PNPO mRNA. (**G**) Live-cell imaging of annotated PNPO with C-terminal

GFP. The localization of annotated PNPO may be altered based on expression level. Cells with high expression of the PNPO Met1 isoform exhibit protein aggregation into foci at high concentrations, whereas low concentrations lead to mitochondrial localization. Scale bar, 5 μm.

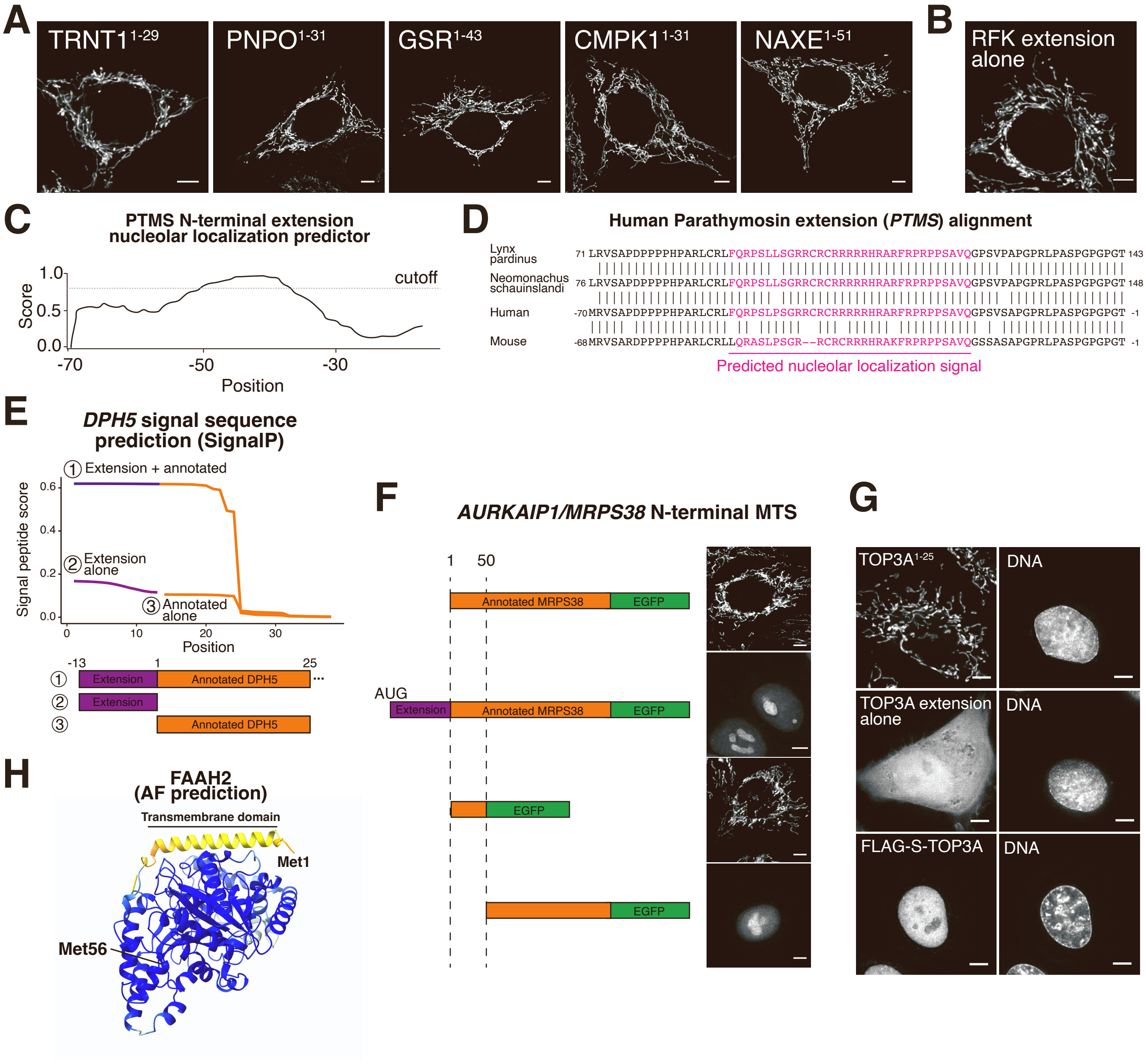

Supplementary figure 3. Mechanisms of N-terminal isoform localization, related to Figure 2. (**A**) Live-cell imaging showing the localization of the N-terminal region of the indicated gene tagged with C-terminal GFP. (**B**) Live-cell imaging of the RFK extension alone (lacks the annotated protein sequence) tagged with C-terminal GFP. (**C**) Nucleolar localization sequence detector [75] output for Human PTMS N-terminal extension. (**D**) Sequence alignment of annotated PTMS from lynx, seal and the N-terminal extension of PTMS from human and mouse. Highlighted in pink is the predicted nucleolar localization signal based on Nucleolar localization sequence detector. (**E**) SignalP prediction [74] for signal sequences for the indicated DPH5 sequences. Combining the N-terminal extension of DPH5 with the N-terminus of DPH5 creates a signal sequence as shown in Fig. 2D. (**F**) Live-cell imaging of the indicated MRPS38/AURKAIP1 construct. (**G**) Live-cell imaging of cells expressing the indicated TOP3A constructs. Scale bar, 5 μm. (**H**) Alphafold prediction of annotated FAAH2, colored by model confidence.

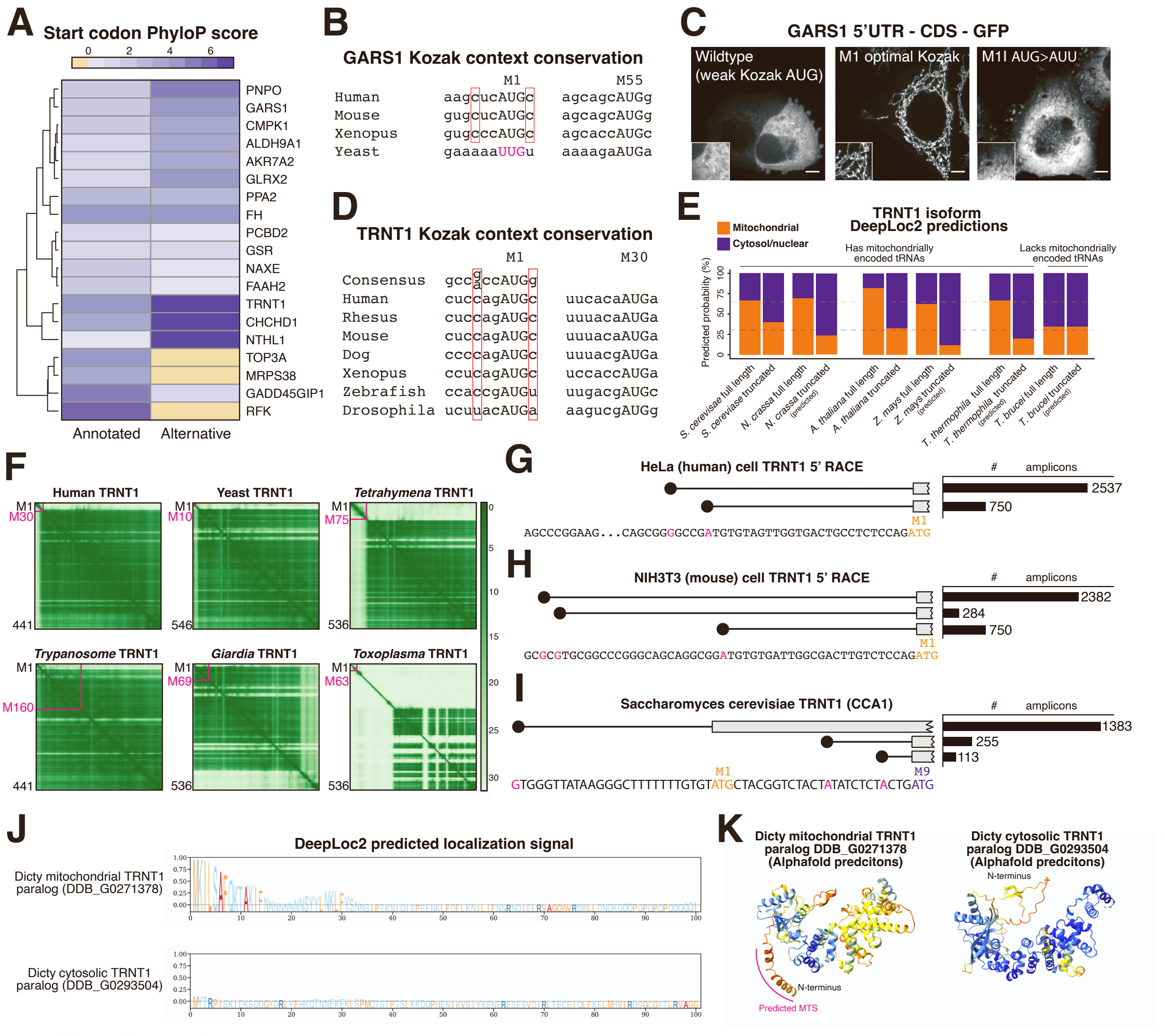

Supplementary figure 4. Evolutionary analysis of alternative N-terminal isoforms, related to Figure 3. (**A**) Heatmap showing the start codon PhyloP score for annotated and alternative ORFs that displayed differential mitochondrial localization. (**B**) Sequence alignment from listed organisms around the GARS1 start codons. The weak Kozak context [79] of GARS1 at the first AUG to allow for leaky ribosome scanning is highly conserved with budding yeast having a non-AUG start codon instead of a weak AUG. (**C**) Live-cell imaging showing the localization of GARS1 5′ UTR – CDS – GFP with the indicated mutations to the first start codon. Scale bar, 5 µm. (**D**) Sequence alignment around the start codons of TRNT1. Consensus sequence is for Vertebrate organisms [79]. The red box is highlighting the -3 and +4 position–particularly important positions for the Kozak context. (**E**) DeepLoc2.1 predictions for TRNT1 isoforms from the indicated organism. For organisms where there is no experimental evidence for truncated TRNT1 isoform, the second in-frame methionine was used. Dashed line indicates average predicted mitochondrial localization probability for *Saccharomyces cerevisae*, *Neurospora crassa*, *Arabidopsis thaliana*, *Zea mays*, and *Tetrahymena thermophila* annotated TRNT1 (orange) or truncated TRNT1

(purple). Similar to *T. gondii* (Fig. 3F), *T. Brucei* lack mitochondrial tRNAs [45] and DeepLoc2.1 predicts only nuclear/cytosolic TRNT1 localization. (**F**) Alphafold predicted aligned error plot for TRNT1 in the indicated organisms. The magenta line indicates the position of the second in-frame AUG start codon coding for the N-terminal nuclear TRNT1 isoform. For Humans, Yeast, and Tetrahymena, the N-terminal truncation removes an unstructured region. For *T. brucei* and *G. lambia*, the predicted N-terminal truncation would remove a large part of the protein's catalytic domain. *T. gondii* TRNT1 N-terminal region is very long and unstructured without and mitochondrial prediction signals. Scale bar indicates expected position error (Ångstrohm). (**G**) 5′ RACE and sequencing from TRNT1 in HeLa (human). The TRNT1 5′ UTR for ensembl ENST00000251607 transcript is shown. Not shown are small number of reads where the 5′ end maps after Met30, which likely represents degradation product or translates to a protein that removes most of the catalytic domain. The magenta nucleotide represents the empirically determined 5′ end in HeLa cells. (**H**) Same as (G) except in mouse NIH3T3 cells. The TRNT1 5′ UTR for ensembl ENSMUST00000113248 transcript is shown. (**I**) 5′ RACE and sequencing from TRNT1 in *S. cerevisiae*. (**J**) DeepLoc2.1 prediction of the two *D. discoideum* TRNT1 paralogs. Note that the next in-frame AUG for the mitochondrial TRNT1 paralog (DDB_G0271378) is Met268, suggesting that encoding two isoforms in this gene is unlikely. Hypothetical usage of this downstream AUG would remove most of the TRNT1 catalytic domain, rendering the protein inactive. (**K**) Alphafold structure predictions for the two *D. discoideum* TRNT1 paralogs, colored by model confidence.

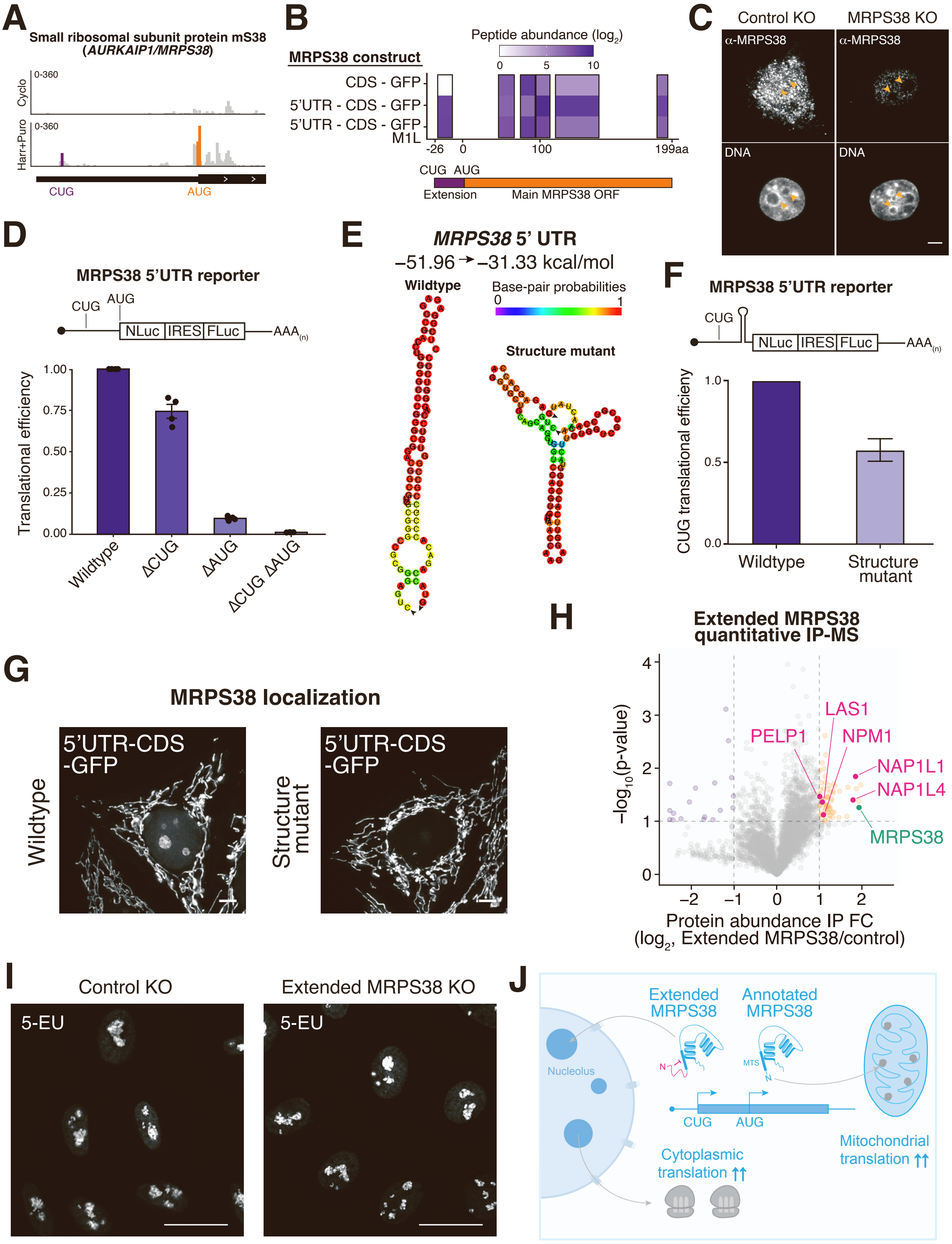

A
Small ribosomal subunit protein mS38
(AURKAIP1/MRPS38)
Cyclo
0-360
Harr+Puro
0-360
CUG
AUG
B
MRPS38 construct
Peptide abundance (log2)
0
5
10
CDS - GFP
5'UTR - CDS - GFP
5'UTR - CDS - GFP
M1L
-26
0
100
199aa
CUG AUG
Extension
Main MRPS38 ORF
C
Control KO
MRPS38 KO
α-MRPS38
α-MRPS38
DNA
DNA
D
MRPS38 5'UTR reporter
CUG
AUG
NLuc IRES FLuc
AAA(n)
Translational efficiency
1.00
0.75
0.50
0.25
0.00
Wildtype
ΔCUG
ΔAUG
ΔCUG ΔAUG
E
MRPS38 5' UTR
–51.96 → –31.33 kcal/mol
Wildtype
Base-pair probabilities
0
1
Structure mutant
F
MRPS38 5'UTR reporter
CUG
NLuc IRES FLuc
AAA(n)
CUG translational efficieny
1.0
0.5
0.0
Wildtype
Structure mutant
G
MRPS38 localization
Wildtype
5'UTR-CDS -GFP
Structure mutant
5'UTR-CDS -GFP
H
Extended MRPS38 quantitative IP-MS
–log10(p-value)
4
3
2
1
0
–2
–1
0
1
2
Protein abundance IP FC
(log2, Extended MRPS38/control)
LAS1
PELP1
NPM1
NAP1L1
NAP1L4
MRPS38
I
Control KO
Extended MRPS38 KO
5-EU
5-EU
J
Extended MRPS38
Annotated MRPS38
Nucleolus
MTS
CUG
AUG
Cytoplasmic translation ↑↑
Mitochondrial translation ↑↑

Supplementary figure 5. Validation, function, and mechanisms of MRPS38/AURKAIP1 N-terminal extension, related to Figure 4. (**A**) Ribosome profiling reads around the MRPS38/AURKAIP1 alternative start codons. (**B**) Mass spec peptide coverage across the MRPS38/AURKAIP1 N-terminal extension for the indicated MRPS38 immunoprecipitation. Constructs with the MRPS38 5′ UTR results in the detection of a peptide mapping to MRPS38 extension. (**C**) Immunofluorescence images of control and MRPS38 knockout cells (both isoform) stained with anti-MRPS38. Orange arrowhead indicates nucleolus. Scale bar, 5 µm. These are polyclonal knockout cells, imaged 6 days post infection, thus residual signal may represent residual protein or background antibody staining. (**D**) Top, schematic of luciferase reporter used to assess MRPS38 start codon activities. Bar plot showing luciferase reporter assays with the MRPS38/AURKAIP1 5′ UTR. The indicated start codons were mutated. Mutation of the extended MRPS38 extension CUG start site reduces nanoLuc activity. IRES-initiated FLuc activity accounts for changes in mRNA abundance and transfection efficiency [39]. (**E**) Predicted RNA secondary structure between the CUG and AUG AURKAIP1/MRPS38 start codons for wildtype (left) and silent structure mutant (right). (**F**) Top, luciferase reporter used to assess the efficiency of the CUG initiated MRPS38 extension start site. Bar plot showing the effect of mutating the RNA secondary structure on MRPS38 CUG translation. N = 3 biological replicates. (**G**) Left, live-cell imaging of wild type MRPS38/AURKAIP1 5′ UTR – CDS – GFP shows dual localization. Right, localization of MRPS38/AURKAIP1 5′ UTR – CDS – GFP with silent mutations that disrupt the RNA secondary structure reduces nucleolar localization (CUG initiated isoform). Scale bar, 5 µm. (**H**) Volcano plot showing quantitative IP-MS analysis of extended MRPS38. Orange points represent proteins that are enriched in the extended MRPS38 IP. Pink points are selected nuclear/nucleolar proteins. N = 2 biological replicates. (**I**) 5-EU incorporation (30-minute pulse) in control knockout (left) and extended AURKAIP1/MRPS38 knockout. Scale bar, 20 µm. (**J**) Schematic highlighting the differential functions of alternative AURKAIP1/MRPS38 isoforms. The annotated isoform functions as a core subunit of the mitochondrial ribosome and the extended isoform localizes to the nucleolus and is important for cytosolic translation.

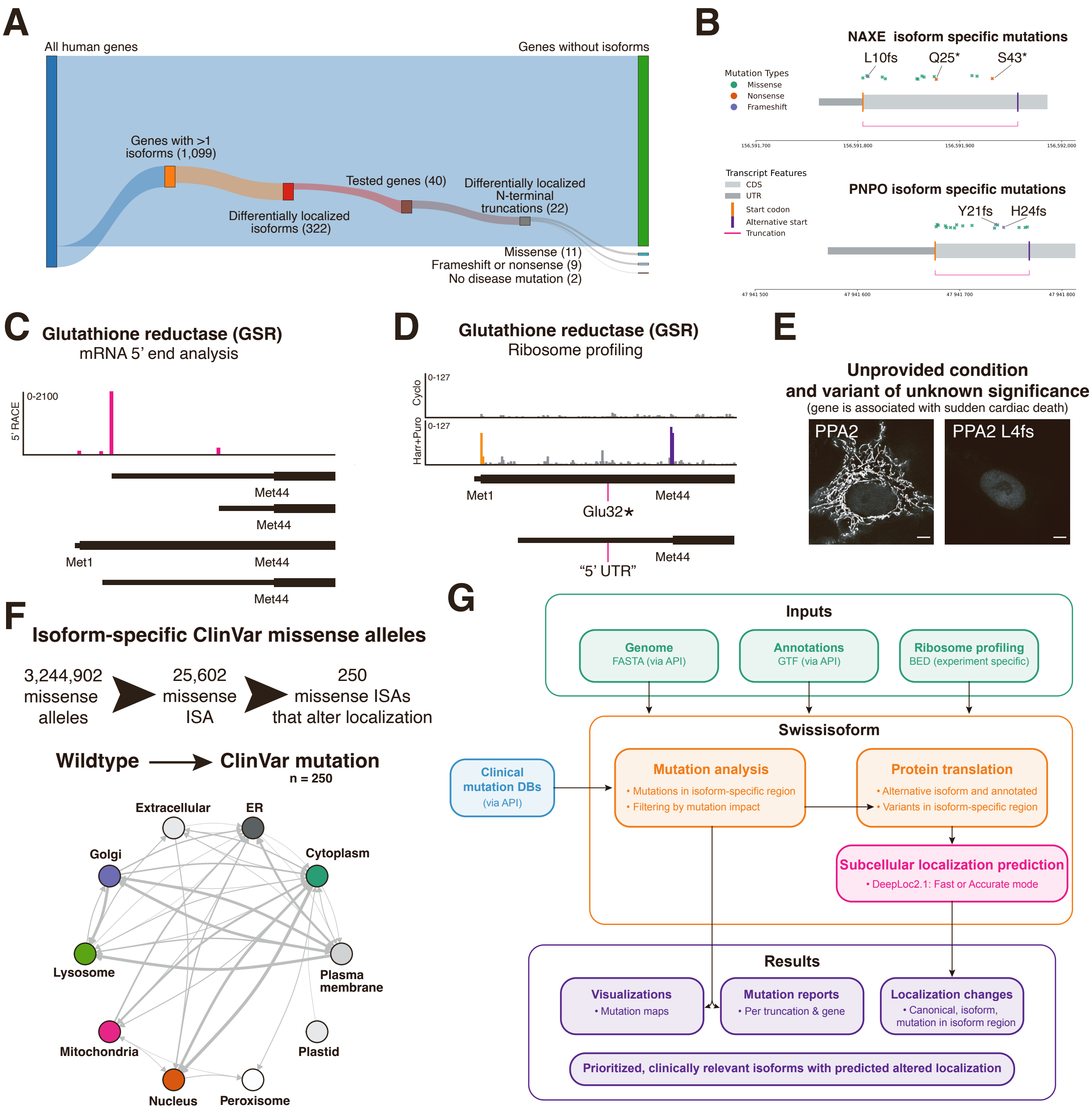

Supplementary figure 6. Pathogenic mutations target specific alternative N-terminal isoforms, related to Figure 5. (**A**) Sankey plot summarizing the number of genes with isoform specific ClinVar mutations. Of the 22 genes that produce differentially localized N-terminal truncations, 9 of them have frameshift or nonsense mutations that eliminate the annotated but keep the truncated isoform intact. (**B**) Examples of genes with isoform specific ClinVar mutations. (**C**) Bar graph showing the number of 5′ end reads from GSR 5′ RACE sequencing showing, suggesting that the alternative GSR isoforms are regulated transcriptionally. (**D**) Ribosome profiling trace around GSR start codons, indicating a pathogenic mutation that is predicted to eliminate full length but not truncated GSR protein isoform. (**E**) Live-cell imaging of wild type of variant of unknown significance L4fs PPA2 5′ 5′ UTR – CDS – GFP. Transfected *in vitro* transcribed reporters were used for this experiment. Scale bar, 5 μm. (**F**) Identification of isoform-specific

missense alleles from ClinVar. Bottom, differential localization predictions comparing wildtype and the ClinVar mutant for the 250 isoform-specific missense alleles. (**G**) Schematic outline of Swissisoform, a platform to identify patient mutations that specifically target an alternative translational isoform.

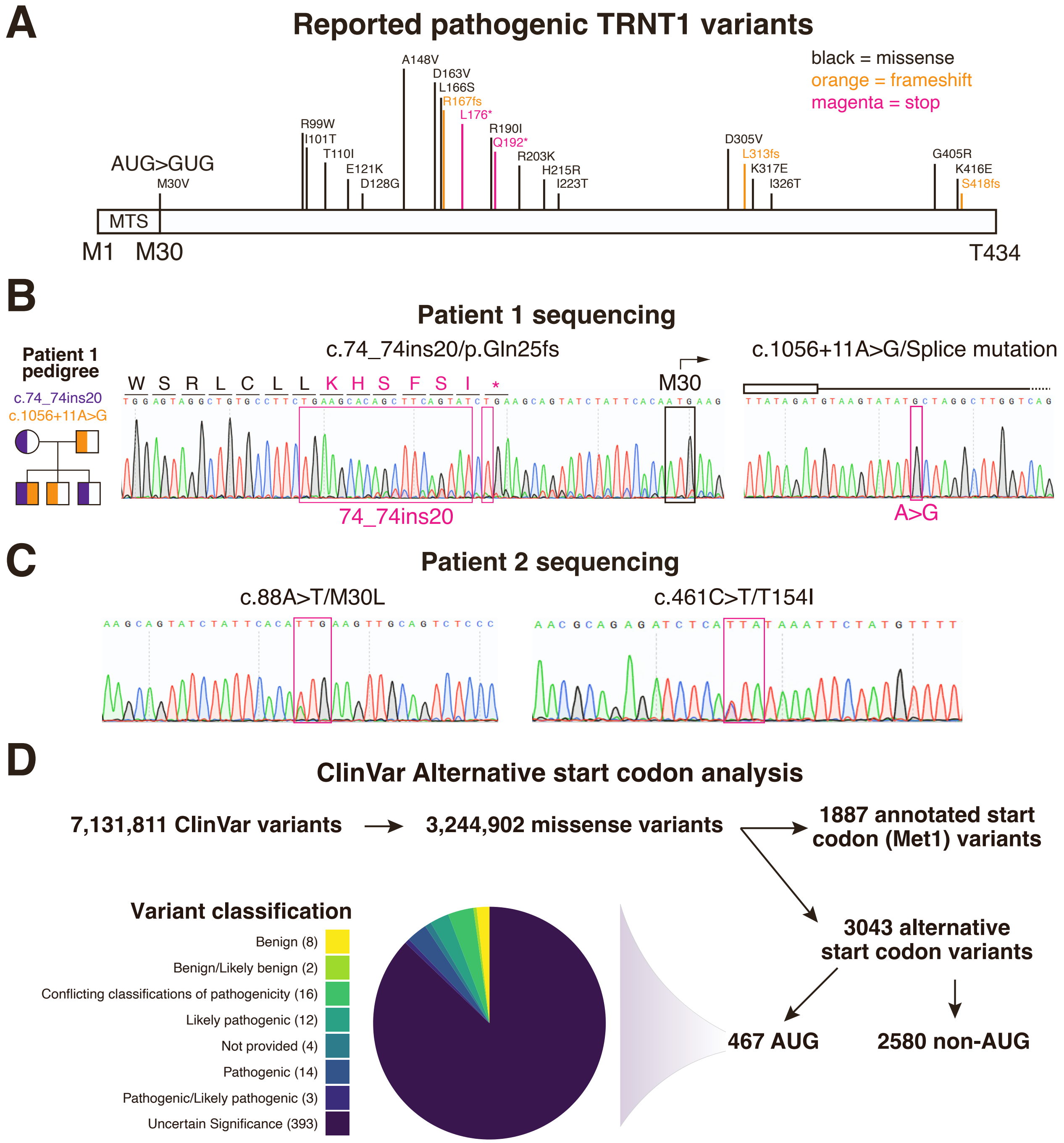

Supplementary figure 7. TRNT1 mutations in SIFD patients, related to Figure 6. (**A**) Reported pathogenic SIFD mutations in TRNT1 from Chen et al.,[63] revealing another truncated TRNT1 specific mutation. (**B**) Pedigree and TRNT1 sequencing data from patient 1. TRNT1 from patient 1 blood samples were subcloned and the Sanger sequencing trace for resulting clones are displayed. (**C**) Sanger sequencing trace for TRNT1 from patient 2 blood samples. (**D**) Analysis of ClinVar to identify "missense" mutations that additionally target alternative start codons.
